# Trends Supporting the In-Field Use of Wearable Inertial Sensors for Sport Performance Evaluation: A Systematic Review

**DOI:** 10.3390/s18030873

**Published:** 2018-03-15

**Authors:** Valentina Camomilla, Elena Bergamini, Silvia Fantozzi, Giuseppe Vannozzi

**Affiliations:** 1 Interuniversity Centre of Bioengineering of the Human Neuromusculoskeletal System (BOHNES), University of Rome “Foro Italico”, Piazza L. De Bosis 6, 00135 Rome, Italy; elena.bergamini@uniroma4.it (E.B.); giuseppe.vannozzi@uniroma4.it (G.V.); 2 Department of Movement, Human and Health Sciences, University of Rome “Foro Italico”, Piazza L. De Bosis 6, 00135 Rome, Italy; 3 Department of Electrical, Electronic, and Information Engineering “Guglielmo Marconi”, University of Bologna, Viale Risorgimento 2, 40136 Bologna, Italy; silvia.fantozzi@unibo.it; 4 Health Sciences and Technologies—Interdepartmental Centre for Industrial Research, University of Bologna, Viale Risorgimento 2, 40136 Bologna, Italy

**Keywords:** magneto-inertial sensors, biomechanics, sports, accelerometer, MEMS, gyroscope, performance assessment, athlete, motion analysis

## Abstract

Recent technological developments have led to the production of inexpensive, non-invasive, miniature magneto-inertial sensors, ideal for obtaining sport performance measures during training or competition. This systematic review evaluates current evidence and the future potential of their use in sport performance evaluation. Articles published in English (April 2017) were searched in Web-of-Science, Scopus, Pubmed, and Sport-Discus databases. A keyword search of titles, abstracts and keywords which included studies using accelerometers, gyroscopes and/or magnetometers to analyse sport motor-tasks performed by athletes (excluding risk of injury, physical activity, and energy expenditure) resulted in 2040 papers. Papers and reference list screening led to the selection of 286 studies and 23 reviews. Information on sport, motor-tasks, participants, device characteristics, sensor position and fixing, experimental setting and performance indicators was extracted. The selected papers dealt with motor capacity assessment (51 papers), technique analysis (163), activity classification (19), and physical demands assessment (61). Focus was placed mainly on elite and sub-elite athletes (59%) performing their sport in-field during training (62%) and competition (7%). Measuring movement outdoors created opportunities in winter sports (8%), water sports (16%), team sports (25%), and other outdoor activities (27%). Indications on the reliability of sensor-based performance indicators are provided, together with critical considerations and future trends.

## 1. Introduction

### 1.1. Background

A successful coaching outcome can be supported by useful and timely feedback to the athlete targeting performance defects. Systematic, objective, and reliable performance monitoring and evaluation, performed by means of qualitative and quantitative analyses of performance variables can reinforce the link between research and coaching practice, especially in elite sports. An alternative to classical laboratory-based assessment is the use of magneto-inertial sensors that can monitor performance without hindering it and with no space limitation or cumbersome setup procedure [1,2,3,4,5,6,7]. These sensors measure physical quantities related to the motion of a body and their measurements can be used to estimate temporal, kinematic and dynamic parameters. New generation sensors permit executing sport tasks during training and performance, are portable, affordable, easy-to-use, and may provide real-time feedback [8]. Recently, the use of such sensors has been revised in swimming [9,10], running [11,12], and strength and ballistic assessment [13,14]. Their partnership with Global Positioning System (GPS) technology has been highlighted for use in team sports [15,16,17,18,19]. In 2015, a systematic review provided a general overview of the dissemination of this technology through different sports disciplines [1]. However, they excluded papers using only sensors mounted on equipment or dealing with non-sport-specific movements (e.g., running). Moreover, the fast expansion of this topic makes any new review quickly outdated. The current systematic review identifies and evaluates updated evidence to answer the following questions: (i) In which sport domains are magneto-inertial sensors currently adopted for athlete performance evaluation? (ii) Are sensor-based parameters used to assess motor capacity (i.e., the highest possible level of functioning of an athlete in exerting maximal velocity or strength in a standardized environment [20]) and analyse the technique of the athlete? (iii) Can sensors be feasibly used to perform match analysis and assess physical demands during training sessions/competitions? (iv) What is currently the potential for the use of inertia-based instrumentation in the field sport setting?

### 1.2. Technology, Requirements and Parameters for Wearable Motion Analysis in Sports

In this section, the necessary background is provided about (i) wearable inertial sensor technology applied in sports to support athlete movement analysis; (ii) requirements to guarantee their accurate use in sport applications; (iii) sensor derived parameters for sport performance assessment.

#### 1.2.1. Gyroscope, Accelerometer and Magnetic Sensors

The advent of Micro-Electro-Mechanical Systems (MEMS) technology has allowed for the manufacture of gyroscope, accelerometer, and magnetic sensors that are highly miniaturized, relatively low-cost, and power efficient.

MEMS gyroscope, accelerometer and magnetic sensors quantify angular velocity, the sum of gravitational and inertial linear accelerations, and the local magnetic field vector components, about and along their sensing axes, respectively. Single-, dual- and tri-axis sensors exist. In the latter case, the three sensing axes are mutually orthogonally mounted, and the aforementioned physical quantities are measured with respect to a Cartesian coordinate system.

The quantities measured by the three sensors can also be used to obtain information about the orientation of a body. However, when the orientation is provided by only one type of sensor at a time, the orientation accuracy is often inadequate for specific applications [21]. Therefore, to improve the accuracy of 2D orientation estimation, gyroscopes and accelerometers are commonly combined in an Inertial Measurement Unit (IMU) [22,23], where the term “inertial” is used because both sensors exploit the principle of inertia to provide either angular velocities or accelerations. When 3D orientation is required, magnetic sensors are also embedded within the Magnetic and Inertial Measurement Unit, widely known by the acronym MIMU (or Inertial and Magnetic Measurement Unit, IMMU, or Inertial and Magnetic Measurement System, IMMS). When IMUs and MIMUs are used, the unit’s orientation is commonly provided as an output of the device, as estimated by combining the data measured by two or three sensors, through means of sensor fusion algorithms [21]. The principles underlying these algorithms, together with a list of parameters that can be derived from IMU and MIMU measurements are presented in Section 1.2.3.

#### 1.2.2. Requirements for Measuring Human Movements in Sport

Several gyroscope, accelerometer, and magnetic sensors, as well as IMUs and MIMUs, have been marketed in recent years for use in human movement analysis. The characteristics of these devices in terms of full-range scale, sampling frequency, power consumption, and portability change considerably according to the application and technology used for data transmission (cabled or wireless). When sporting applications are targeted, care must be taken in order to properly select the measurement system, according to the characteristics of the selected motor task and to the variables that are estimated (e.g., accuracy requirements for trajectory determination in sports [19]). This aspect ranked first in the identification of user requirements for coaches, followed by the request for repeatability, understandable and communicable feedback to athletes, real-time results, suitability for multi-athlete analysis, and non-interference with the athlete or coach [24]. In general, the system used should also be small and light (portable, suitable for in-field use); wearable (wireless); easy and quick to set up and analyse; usable on any surface and in any environmental condition (including under water through hermetic sealing and limited drag, if swimming applications are targeted); power efficient (in case long-time monitoring is required, e.g., marathon monitoring); and have adequate full-range scale and sampling frequency. The latter should be determined according to the motor task, parameters of interest, and measurement system, being a minimum of two times the highest frequency in the signal of interest (according to the Nyquist–Shannon sampling theorem [25]), to minimise the loss of relevant information [13].

Besides the characteristics of the selected device, users must be aware that the signals derived from these sensors are affected by different sources of error which, even if compensated for, currently limit the possible range of applications. Among them is gyroscope and accelerometer bias, caused by changes in the MEMS physical properties and leading to drift errors that increase linearly or quadratically over time when the measured signal is integrated once (to obtain orientation) or twice (for position estimation), respectively [26]. Secondly, the oscillations between body and device, i.e., soft tissue artefacts [27], compete with drift as the most crucial source of error. The fixing technique may limit the oscillations as their compensation is essential [28]. However, as soft tissue artefacts are motor task- [29] and subject-dependent [30], as well as sensitive to the site and method of the unit attachment [31,32] and to unit removal and replacement [33], their compensation is very critical. Moreover, they can be assumed to be larger during explosive tasks; to analyse such tasks, the device mass should be limited to a minimum. Thirdly, ferromagnetic disturbances (particularly indoors) may come from ferromagnetic objects or electrical appliances like, for example, water pumps under the pool. They alter the magnitude and direction of the measured magnetic field vector, thus causing errors in the estimated orientation [34].

#### 1.2.3. Sensor Derived Parameters

Gyroscope, accelerometer, and magnetic sensor data can be used to estimate different parameters. These could be based on the detection of features in the measured signals or on more sophisticated processing techniques, which, for example, enable information provided by two or more sensors to be combined. Sensor derived parameters can be divided into three main categories: temporal, kinematic, and dynamic.

##### Temporal Parameters

Temporal parameters are typically obtained by detecting features (e.g., maxima, minima or slope changes) in the measured angular velocity or acceleration signals. Some examples of these parameters are: (i) time instants related to the beginning or end of a movement, foot or hand contact, ball release or other events of interest; (ii) phase identification and segmentation of sport tasks using the time instants; (iii) time intervals, such as stride/step/stance duration in locomotion or stroke duration in swimming; and (iv) frequency in cyclic movements.

##### Kinematic Parameters

Kinematic parameters refer to both angular and linear forms of position, velocity and acceleration. In particular, the estimation of the 3D orientation of a MIMU with respect to an inertial reference frame is of fundamental importance as it is essential when obtaining either the absolute orientation of body segments or sports equipment (e.g., rackets, clubs, skis), or the orientation of a body segment relative to another (joint kinematics). Moreover, it is necessary to set apart the gravitational and inertial components of the linear accelerations, in order to estimate the linear kinematic parameters, such as MIMU position or linear velocity.

The 3D orientation of a MIMU may be estimated by numerical time-integration of the differential kinematic equations relating the time derivatives of the orientation parameters to the angular velocity provided by the gyroscope. The accuracy of this numerical integration, however, is hindered by errors that increase with time due to gyroscope drift [21,26]. Moreover, the initial conditions of the integration process must be determined. To manage these problems, the signals provided by the accelerometric and magnetic (aiding) sensors are used under the following hypotheses: (i) the accelerometers measure only the gravity vector, true only when the MIMU moves at constant speed or is stationary; (ii) both the gravitational and magnetic field vectors are uniform and constant within the measurement volume, which, as mentioned above (Section 1.2.2), is not necessarily the case for the magnetic field vector. Therefore, the information provided by the three sets of sensors is combined within a sensor fusion framework with the aim of proficiently exploiting all the available information. Examples of sensor fusion approaches are stochastic filtering, often implemented in the form of an Extended Kalman Filter [21,35] and complementary filtering [36,37].

Once orientation is obtained, joint kinematics can be estimated as the relative orientation between adjacent body segments. Its computation requires the definition of a multibody kinematic model, together with its anatomical calibration, and the determination of the reference “zero” joint configuration [38]. Among other angular kinematic parameters, the angular velocity is often exploited as it is directly measured by gyroscopes, while the angular acceleration must be obtained through numerical differentiation of the measured velocity. Like the integration process, computing the derivative of a discrete signal entails errors resulting from the differentiation method and the type of representation used for numbers (precision level) [39].

As for linear kinematic parameters, in theory, the measured linear acceleration can be numerically integrated, once amended by its gravitational component, to obtain an estimate of the linear velocity and, if further integrated, the displacement (trajectory). Again, the initial conditions of this process are required, and drift errors may occur and propagate due to low-frequency gyroscope drift. Moreover, the removal of the gravitational component is prone to errors related to the orientation calculation. For these reasons, model-based approaches are often required [40] and the accurate estimation of linear position from MIMUs still represents a challenging and time-consuming exercise [21,26].

It is noteworthy to specify that centre of mass (CoM) parameters estimated from the acceleration of a single sensor positioned on the lower trunk, nearby the position of the CoM in the static upright posture, is an approximation that does not take into account the dynamics of all the body segments, specifically the lower and upper limbs [41]. Still, this information can be useful in in-field sport performance evaluation without requiring cumbersome setup.

##### Dynamic Parameters

Like kinematic quantities, dynamic parameters refer to both linear and angular variables. Although, in theory, vertical/leg stiffness, external forces, joint forces, moments, and powers can be estimated from MIMU measurements, the accuracy and reliability of these parameters are contingent on the accurate estimation of kinematic variables (e.g., temporal parameters, orientation, and position). Additionally, the embedding of measures of external forces in hybrid inverse dynamic methods is recommended to reduce errors of pure inverse dynamic estimates of joint loads [42,43].

## 2. Materials and Methods

### 2.1. Inclusion and Exclusion Criteria

Using the PICOS framework (which focuses on Population, Intervention, Comparison, Outcomes, and Study design [44]), the selection criteria and search strategy were designed to answer the above-mentioned research questions. Full papers published in English, including studies using MIMUs and IMUs to analyse motor tasks of interest in a sport context, performed by recreational or elite athletes, were considered. During the final screening, selections were made based on the relevance of the identified sources to the assessment of maximum strength and ballistic performance, technique related parameters, and intensity measures for match analysis. The other inclusion and exclusion criteria are described in Table 1.

### 2.2. Search Strategy

The review of the literature was performed by selecting articles from Web of Science, Scopus, Pubmed, and Sport Discus databases, published in English, until 12 April 2017. Keywords were selected to define the measurement instruments, the sport activity performed, the participant tested and to exclude studies dealing with patients, risk of injury, and the assessment of physical activity or energy expenditure (the Boolean research strategy used is reported in Table 2). A keyword search was performed to match words in the title, abstract, and keyword fields. Electronic searches were performed by one author (E.B.); references and abstracts were stored alphabetically into a separate worksheet. Additional relevant papers were thereafter selected by examining the reference lists of selected papers.

### 2.3. Review Process

Conference proceedings, theses, and duplicate journal references due to searches in different electronic databases were removed first. During the subsequent screening, selections were based on the relevance of the identified sources to the inclusion criteria. Two independent reviewers (E.B. and V.C.) evaluated titles and abstracts of the retrieved papers for inclusion. A full text evaluation was then performed if the title and abstract failed to provide adequate information. Any disagreement between the two reviewers was resolved by a consensus meeting of all authors.

## 3. Trends

### 3.1. Paper Selection and Identification

**Figure 1:**
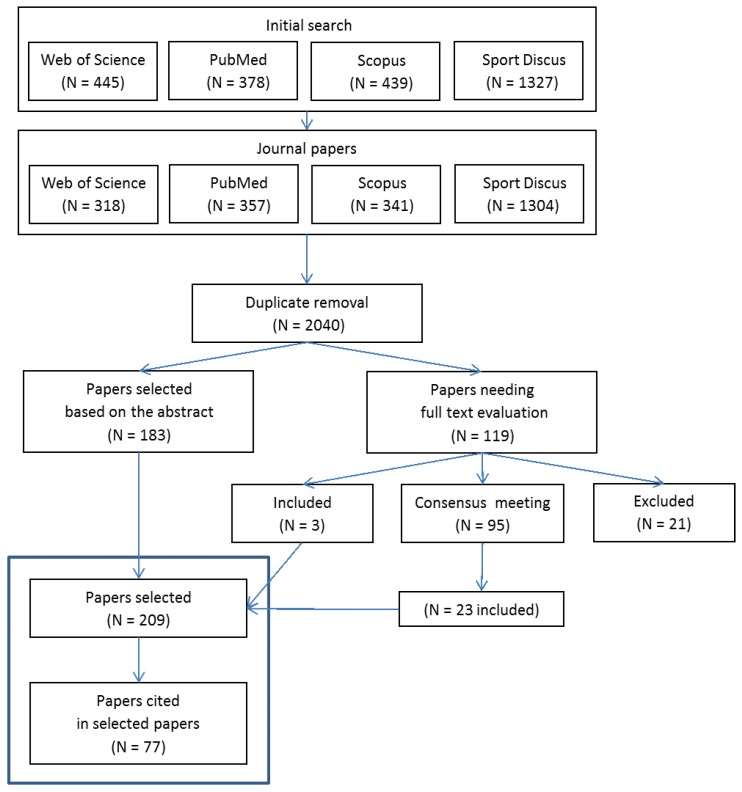
Search strategy flow chart. After the removal of duplicates, 2040 papers were screened for inclusion: 183 were selected based on title and abstract, 119 full texts were further analysed. Three were included directly and 23 out of 95 were included after a consensus meeting. The references of the 209 papers obtained from the database search and those cited in the 23 reviews included in this paper were screened leading to the inclusion of further 77 papers for an overall total of 286 papers.

The duplicate removal yielded 2040 citations (Figure 1). One hundred and eighty-three papers were included based on title and abstract, whereas the full text was reviewed for 119 papers. Three papers were directly included, while 95 papers were discussed during multiple consensus meetings, leading to the inclusion of a further 23 papers. Several studies were excluded for not involving athletes. Fourteen review papers were retrieved from the search [1,3,4,5,9,10,11,13,16,17,18,19,45,46] and were analysed separately, along with nine additional reviews cited by the selected papers and reviews [2,12,14,15,47,48,49,50,51]. Screening of the selected papers and the 23 reviews produced 77 additional papers. As a result, 286 studies were identified for inclusion in the systematic review [24,41,43,52,53,54,55,56,57,58,59,60,61,62,63,64,65,66,67,68,69,70,71,72,73,74,75,76,77,78,79,80,81,82,83,84,85,86,87,88,89,90,91,92,93,94,95,96,97,98,99,100,101,102,103,104,105,106,107,108,109,110,111,112,113,114,115,116,117,118,119,120,121,122,123,124,125,126,127,128,129,130,131,132,133,134,135,136,137,138,139,140,141,142,143,144,145,146,147,148,149,150,151,152,153,154,155,156,157,158,159,160,161,162,163,164,165,166,167,168,169,170,171,172,173,174,175,176,177,178,179,180,181,182,183,184,185,186,187,188,189,190,191,192,193,194,195,196,197,198,199,200,201,202,203,204,205,206,207,208,209,210,211,212,213,214,215,216,217,218,219,220,221,222,223,224,225,226,227,228,229,230,231,232,233,234,235,236,237,238,239,240,241,242,243,244,245,246,247,248,249,250,251,252,253,254,255,256,257,258,259,260,261,262,263,264,265,266,267,268,269,270,271,272,273,274,275,276,277,278,279,280,281,282,283,284,285,286,287,288,289,290,291,292,293,294,295,296,297,298,299,300,301,302,303,304,305,306,307,308,309,310,311,312,313,314,315,316,317,318,319,320,321,322,323,324,325,326,327,328,329,330,331,332,333,334] (Figure 1) and analysed along with the 23 reviews in the following sections. Details of the papers’ aims, performance evidence, sport, motor task, participants, setting, parameters, device characteristics and fixing are reported in the Supplementary Material.

### 3.2. Journals and Years

**Figure 2:**
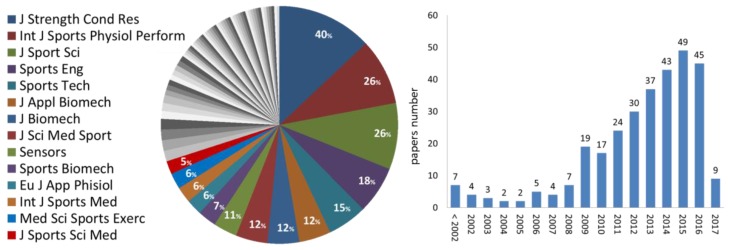
Distribution of the reviews and papers in the systematic review over journals (in %) and time (in absolute values).

The papers included in the systematic review (286 papers) and the reviews (23 papers), (total: 309 papers) were published in 88 different journals, 71% of which appeared in 14 journals, each publishing at least five papers (Figure 2). Trends of publication along the years are also shown (Figure 2).

### 3.3. Participants, Setting, Sport, Motor Task

The papers included in the systematic review (286 papers) analysed elite (declared as such, professionals, or national to international level, 38% of the papers), sub-elite (competitive, university, highly trained, and experienced athletes, 21%) and recreational athletes (26%), while multiple experience levels were included in 8% of the papers, and 7% were not specified. Three studies involved Paralympic athletes [124,125,126] and two involved wheelchair junior athletes [71,292]. Studies analysing athletes in the field during training or simulated training comprised 62% of the total analysed, with 7% undertaken during competition, and 28% of the studies performed in laboratory settings. When multiple settings were included in a single study, the setting closest to performance was counted.

As detailed in Table 3, cyclic and team sports comprised 35% and 25% of studies analysed, respectively. The possibility of measuring movement outdoors created opportunities in winter sports (aerial skiing, alpine skiing, cross-country skiing, ski jumping, ski mountaineering, snowboarding: 8%), water sports (diving, swimming, rowing, kayaking: 16%), team sports (American football, Australian football, baseball, basketball, contact sports, cricket, field hockey, Gaelic football, handball, ice hockey, netball, rugby, soccer, softball, volleyball, wheelchair basketball: 25%), and other outdoor activities (sprint and distance running, cycling, golf, tennis, rock climbing, shot-put, roller skiing, skateboarding: 27%).

Regarding the specific motor tasks investigated, almost all belonged to the following broad gross motor skill classifications:

Locomotion:

- aerial movements (diving trampoline jumps, half-pipe snowboard, front/back somersaults)
- agility runs (change of direction, side-cuts, turning)
- bicycling (indoor or outdoor track, jumping)
- horse riding (trot, gallop, circles, change of directions)
- jumping (with and without counter movement, with one or two legs, drop landing, drop jump, stop jump, jump header, moving header, standing short\long jump, springboard)
- climbing
- swimming (starts, front crawl, backstroke, breaststroke, butterfly, freestyle, turns)
- roller skating and skateboarding
- rowing and paddling (single and double scull rowing, kayak propulsion)
- running (treadmill and over ground, distance and sprint running, hurdles)
- skiing and snowboarding (giant slalom, downhill, cross country runs, ski jumping, uphill ski mountaineering, roller skiing)
- wheelchair sprint and turns.

Collisions, takedowns, or weightlifting:

- tackling and sustaining physical collisions
- takedowns
- weightlifting, entailing the use of upper or lower limbs (snatch lift, back squat, pull/throw on bench press).

Throwing, aiming, or hitting:

- kicks (soccer in step kick, karate front kick)
- trunk rotations (golf and baseball swing, ice hockey slap shot, baseball bat swing)
- overarm movements (tennis serve/volley/strokes, pitching, cricket bowling, javelin throwing, shot put) sidearm and underarm movements (air pistol shots, boxing straight punch, karate jabs/crosses/hooks/upper-cuts, fencing lunge and touche, bowling ball throw, golf putt).

### 3.4. Technique Analysis, Intensity Measures for Match Analysis, Motor Capacity Assessment, and Activity Classification

In the revised literature, the primary focus was technique analysis (55.5%), followed by match analysis (20.7%), and capacity assessment (17.3%), with few papers dealing with activity classification (6.5%). Papers are presented as classified into these four categories and referenced in more than one category when appropriate.

#### 3.4.1. Technique Analysis (163 Papers)

**Figure 3:**
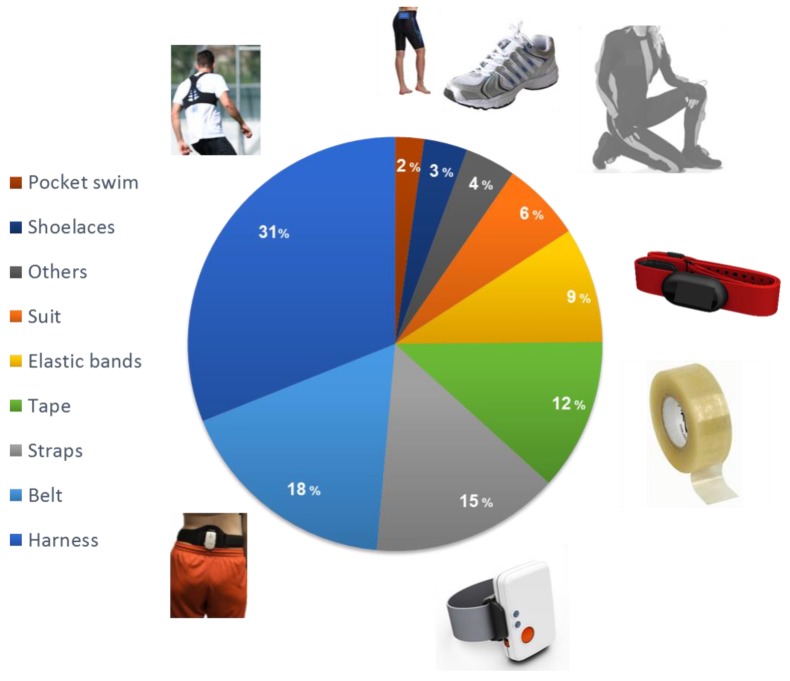
Device fixing mean in percentage (%) of the total papers, excluded those not specifying the fixing modality.

Spatio–temporal parameters (phase identification, foot contact timings, stride/step/stroke number, duration, frequency), linear and angular kinematic variables (position/orientation, mean or instantaneous linear/angular velocity, acceleration), and linear and angular dynamic parameters (leg stiffness, joint forces, moments, powers), listed in Table 4, were estimated based on the many possible configurations of sensor fixing on athletes and/or sport equipment depicted in Figure 3.

##### Spatio–Temporal Parameters

For non-cyclic tasks, temporal parameters were identified to measure critical temporal events (blade–puck contact time in ice hockey [206,309], cricket bowling [227] and, more specifically, ball release [288]), or detect task phases and critical events (in ski jumping [90,91], half-pipe snowboard [159], bowling [180], baseball swing [146,179], instep kick [228], karate front kick [273], diving trampoline jumps [161], artistic gymnastics springboard jumps [187], golf [171], javelin throw [270], soccer turning manoeuvres [241], swimming tumble turn [192,197,285] and start [193] and cricket bowling [264]).

For cyclic tasks, temporal identification can entail the simple identification of a stride/step/stroke event to measure their number, rate, duration, or the more complex identification of specific events within the cycle to subdivide it into different phases. Stroke duration and/or number has been identified in kayaking [260], single sculler [116] and multi-person rowing [217,275], and swimming. For swimming, sensor placement at the trunk/limbs/head was used to provide either arm strokes (for front crawl [63,80,107,110,263]), kick strokes (for front crawl [124,126] and freestyle [125]), or generic strokes (for front crawl [24,68,85,108,156,164,168,263,323,332], butterfly [24,68,85,156], breaststroke [24,68,85,164,291]). Stride and step frequency/duration have been assessed during running [153,157,160,162,174,191,202,204,229,230,242,243,262,294,295,302,321,327], skating [293], and in the run up of cricket ball delivery [264]. Revolution rate was characterised for a bowling ball [180] and during cycling [302,313]. Phase segmentation was performed in swimming (to identify front crawl stroke phases [80,164,246,247]), in cross-country skiing [120,207,237] and uphill mountaineering [121] (to determine cycle rate from plant and lift-off of each ski and pole ground contact), in ice hockey skating (to determine cycle rate from initial contact to blade-off for each skate [293]), and during running on a track (trunk placement [61,149,153,194] and shank or foot placement [140,209,295]), on a treadmill (trunk placement [77,203,315,320] and shank or foot placement [87,140,148,222,229,230,294,318]), and during the acceleration/maintenance phase of sprint running (trunk placement [70] and foot placement [57]).

Corresponding to these temporal parameters, the average step/cycle length was obtained for arm strokes in swimming [63,108,156] and the average stride length was derived from either measured motion and contact times (for treadmill running [87,229,230,294] and track running [327]), and dividing the average cycle velocity by cycle time (in running [328], cross-country skiing [120,237] and uphill mountaineering [121]).

##### Body Segment and Centre of Mass Kinematics

Obtaining *body segment* and *CoM instantaneous velocity* from the integration of acceleration in the global frame entails the challenge of properly compensating for sensor drift through the design of appropriate processing techniques. These algorithms first remove gravity from acceleration data (see below for orientation estimation methods) and then integrate acceleration by the following method:

- using known treadmill motion information (during simulated cross-country skiing [41,237])
- performing the integration over a short time duration through proper segmentation of the task and using a priori information to reset the error at each cycle (running speed obtained from a shank sensor [328])
- assuming equal initial and final conditions for vertical velocity and position (during vertical jumping [220])
- segmenting the data and removing mean velocity, under the assumption that each temporal segment is at steady-state (mean velocity computed by averaging few rowing cycles with a moving window for single sculling [116], or according to changes in forward acceleration variance for swimming [106])
- through data fusion with GPS based velocity estimates (during running and cycling [302], alpine skiing [122], and snowboarding [333]).

Alternative to the use of measured acceleration, body segment or CoM velocity can be derived from the corresponding angular velocity and definition of a forward kinematic model (used to estimate forward speed of the tennis racket head [54] or for full body CoM during alpine skiing [122]). Subject specific models were also developed to estimate CoM velocity during running, based on step rate measures [242]. The errors entailed in instantaneous velocity estimation can be partially overcome by limiting the velocity analysis to average values in cyclic sports, for example, for each swimming stroke [104,105,106,108,290] turning action [192], lane [63,80,323], running cycle [162,328], or mean velocity in running [56,160]. The average velocity of progression was also obtained from ski IMUs, removing drift and assuming zero-velocity during a portion of the ski thrust phase, in cross-country skiing [120,237] and in uphill mountaineering [121].

Obtaining *forward horizontal* and *vertical CoM displacement* is less sensitive than estimating velocity due to inaccuracies in the removal of gravitational acceleration and to drift errors amplified by the double integration of acceleration. Such variables have been determined under two possible conditions: either when drift errors were small with respect to the measure of interest, or when displacement was obtained with respect to a known reference position [315]. The former applies, for example, to the assessment of large displacements from a starting position, such as during jumps when skiing, snowboarding, mountain biking (height and horizontal distance obtained using GPS data) [266], cross-country skiing [41,237], and uphill mountaineering [121]. Error can even become negligible when CoM global trajectories are used solely to support the interpretation of other estimated variables, as is done for external force estimates during ski racing [75]. Six possible approaches have been proposed to derive known reference positions:

- subtracting the linear trend line over the analysed time period from the Euler angles [41]
- assuming that the mean value is constant for cyclic events, to derive vertical CoM excursion from peak to peak values within each cycle (while running on a track [295] or on a treadmill [155])
- defining a body model and measuring body segment orientation with respect to a reference to obtain vertical and horizontal CoM displacements (with respect to the skis at take-off during ski jumping [88], with respect to the ground during a golf swing [238] and with respect to a Global Navigation Satellite System (GNSS) antenna placed over the head during alpine skiing [122])
- starting and ending the movement in known positions, to obtain shoulder, elbow and wrist [185], and solely elbow trajectories [267] during baseball pitching
- through data fusion with GPS based position estimates, which simultaneously compensates for short-term GPS outages (during outdoor activities such as snowboarding [333])
- fusing the velocity of a root point (integral of a pelvic sensor acceleration) with the velocity estimated from the lower limb kinematics at ground contact [40,335].

*CoM acceleration* was also assessed to detect *event* timing, typically the impacts during running (from the upper body [72,295], heel and metatarsal heads [148], and the tibia [140]). Impact *amplitude* was reported for running [140,325], landing from horizontal jumps [248], and for tennis racket shocks [59]. Variations in acceleration amplitude are also considered relevant when characterising running performance [56,174,200,201,203], running economy (acceleration root mean squared value divided by average speed) [224,276] and symmetry [184,191,196], as well as turning manoeuvres in soccer [241].

*Body segment kinematics* were considered to assess various parameters: postural tremor of air pistol shooters [303]; take off velocity and angle during standing horizontal jumps as obtained from body segment orientations [248]; shank linear and angular velocity, angular acceleration, and antero–posterior linear acceleration during an instep kick [228]; tibial accelerations in depth jumps [283]; heel lift and forearm acceleration to describe running economy [295]; jerk of hip trajectory in climbing [279,280]; and segment vibrations during road and off-road cycling [212] and, in the latter condition, with different wheels [211].

##### Body Orientation

*Body segment orientation* was usually estimated by fusing data from different sensors, for example, using MIMU data alone to estimate trunk inclination during a sprint start [69], running on a track [295], during a golf swing [238,314], and during outdoor activities, such as snowboarding [333]. Inclination of body segments and angular velocity about the cranio–caudal axis were also estimated during trampoline jumps [161]. Pelvis orientation was dealt with in swimming [192] and climbing [279]. MIMU data were complemented by seat reaction forces to estimate bike and rider trunk orientation [330], while GPS data were added to estimate limb orientation in ski racing [75]. Constraints specific to a task, such as known starting and ending positions, were used to improve the accuracy of upper limb orientation during running [295] and baseball pitching [185,267]. Similarly, aerodynamic constraints were embedded within a sensor fusion algorithm to estimate sacrum, thigh, and shank orientation during ski jumping [88,89]. Few studies estimated the trunk rotation about a longitudinal axis: one study hypothesised that the accelerometers measure only the gravity vector, i.e., the acceleration directly provides the sensor orientation (swimming [63]) whereas another study used numerical time-integration of gyroscope data to analyse aerial manoeuvres in half-pipe snowboarding [159]. The latter approach was also used to obtain upper arm internal rotation angles in tennis [53,54]. A pioneering application about body orientation using IMUs was given in the definition of human whole body pose during bicycle riding by considering the constraints characterising human–bicycle interaction [331].

Estimating body segment orientation is also essential when *removing the gravity vector* from measured accelerations to assess CoM acceleration in the global frame. In this framework, drift removal was performed either through data fusion (alpine skiing [189]) or, if all sensors were not available, under task-specific assumptions. For example, during swimming, the average axis about which the sacrum rolls was assumed to be in the forward direction [106] or during running, yaw was assumed to be zero when moving in a straight line [302] or, again, that the net measured acceleration of the runner was equal to gravity and that its orientation was constant during the trial [155].

*Joint kinematics* require both a good orientation estimate and appropriate anatomical calibration. They were determined for wrist flexion (tennis serve [54], swimming [119]), shoulder angular displacements (internal-external or/and abduction-adduction: tennis serve [53,54], internal–external rotations in running [295], flexion–extension, abduction–adduction, and internal–external rotation in swimming [119]), elbow angular displacements (flexion–extension and pronation–supination in swimming [119]), hip angular displacements (flexion–extension in ski jumping [90], flexion–extension, abduction–adduction, and internal–external rotation in cycling [96], external rotation in horse riding [138]), hip angular velocities on the sagittal plane (karate front kick [273]), knee angular displacements (flexion-extension in ski jumping [90], alpine skiing [187], cycling [214] and snowboarding [188], flexion–extension, abduction–adduction, and internal–external rotation in alpine skiing [333]), knee angular velocity on the sagittal plane (karate front kick [273], running [295]), shank–ski angle on the sagittal plane (ski jumping [90]), ankle angular displacements (plantar–dorsiflexion, inversion–eversion, and internal–external rotation in soccer specific tasks [55], plantar–dorsiflexion in cycling [214]), and for all the main joints during trampoline jumps [161].

##### Object orientation and kinematics

The estimation of these parameters entails coping with the typical sensor errors but does not entail soft tissue wobbling. Orientation and kinematics have been estimated for several activities:

- alpine skiing (ski inclination in a lateral direction with respect to the Earth’s fixed coordinate system, describing the skis’ steering and edging manoeuvres [189,231])
- baseball (bat position, orientation, linear velocity during the swing [179]; ball’s velocity at release [221])
- bowling (ball velocity [180])
- combat sports (acceleration of the boxing bag at the time of the punch [79,239,284])
- cricket bowling (outward acceleration at ball release [288])
- cycling (roll angle [330], crank angle [95])
- ice hockey (puck velocity [309])
- fencing (foil speed during lounge and *touche* [329])
- golf (clubface orientation, head velocity, and position [181]; clubface orientation, head velocity and acceleration [171])
- gymnastics vaulting (springboard kinematics during jump on and take-off [187])
- javelin throw (javelin kinematics during the throw [270])
- kayaking (instantaneous boat velocity [169])
- rowing (boat acceleration [81,205,260,275,287], orientation [287], velocity [217,287], forward movement and balancing, propulsion, and distance travelled [217], as well as boat–oar stroke angle [205,287], oar acceleration [205]), seat position [287])
- shot-put (shot acceleration [139])
- ski jumping (ski velocity [300], ski horizontal and V-opening angles during jumping [90])
- softball (ball’s velocity at release [221])
- tennis (consistency of backhand, forehand, overhand swings [301])
- outdoor running (path incline [162])
- uphill mountaineering (ski slope orientation [121])
- weightlifting (barbell acceleration measured during power snatch, power clean and jerk from the rack [123] and snatch lift [271]).

In some studies, reference values for MIMU data integration were used to estimate instantaneous velocity (GPS data for kayak analysis [169], video cameras for javelin kinematics during the throw [270]).

##### Dynamics and Power

The estimation of dynamic variables is rarely carried out, due to difficulty in assessing external forces. None of the analysed studies carried out a validation against dynamic measures, but some studies reported data from the literature for comparison [43,75,88]. *External forces* were estimated through inverse dynamics; total aerodynamic force applied on the athlete’s body assumed as rigid, during the stable flight phase of ski jumping [88]; hand force applied on the javelin during the acceleration phase of the throw [270]; force and time duration of a strike on a punching bag [79,239,284]; centrifugal force, applied outward on a skier during a turn [231]. To estimate forces exchanged with the environment during skiing, inverse dynamics requires CoM acceleration, estimated from an articulated system of rigid bodies (using a body model). The resultant force acting on the athlete’s CoM, perpendicular to the ski table, was obtained during the take-off phase of ski jumping [88]. For overground skiing, information about the ratio of loading between the skis is also required to resolve the indeterminacy of the inverse dynamics problem. An approximation of this ratio was obtained from pressure sensor insoles embedded into the ski boots, in order to estimate the ground reaction force applied to each foot, aerodynamic force, ski friction, power, and energy during ski racing [75]. *Joint dynamics* were obtained through pure inverse dynamics for tasks entailing no exchange of forces with the environment, as is typical for open kinematic chain movements of the upper and lower limbs (elbow forces in baseball pitching [158] and shank dynamic variables during instep kicks [228]). For tasks entailing the exchange of forces with the environment, dynamic measurement systems and a hybrid inverse dynamics approach were used, mounting force plates on the snowboard, to obtain lower limb net joint moments during snowboarding turns [43]. IMUs were also used to obtain *vertical stiffness* during running, by estimating the vertical ground reaction force during the support phase using measured contact times and the participant’s body mass [77].

##### Spectral Analysis

The frequency content of the acceleration of body segments, usually analysed in relation to the risk of injury, was, in some cases, associated with performance in (i) runners, when investigating the association between strike patterns and shock attenuation [157,229,230] or soft tissue vibrations [118]; (ii) air pistol shooters, during aiming, as postural tremor is associated with performance [303]; and (iii) cross country mountain biking, to describe the relationship between vibration mechanics and their interaction with the terrain, bicycle, and rider [211].

##### Technique Analysis Objectives

The objectives related to technique analysis pursued with MIMUs span a broad spectrum, including

- visual inspection of kinematic data to support qualitative understanding of the technique (kayaking [260], running [321], springboard diving [173], weightlifting [272], cross-country skiing [215])
- pacing strategy, determination by stride/step/stroke rates assessment (detailed references in the spatio–temporal parameters section)
- assessment of inter-segment coordination (lower-limbs during the take-off extension in ski jumping [89], hip, chest, and wrist during baseball swings [146]) and of inter-arm coordination (front-crawl [107,108])
- assessment of symmetry (running gait [184,191,196], wheelchair propulsion [71], horse riding [138]) and regularity\variability (running gait [223], cycling [276,313])
- detection of foot strike type [148,295]
- assessment of the impact of training tools and accessories during running (static stretching [209], wearing graduated lower-leg compression sleeves [294]) or of caffeine supplementation during simulated field hockey [112]
- comparison of different techniques (cross-country skiing [215,237])
- evaluation of the adherence of a movement technique to appropriate normative data (performance model) for both injury management and performance enhancement [52]
- investigation into the mechanisms that maximise speed in multi-articular joint movements (increase in muscular efficiency vs different muscular activation patterns in fencing [329] and karate [273])
- investigation into the effect on performance of varying the sports objects characteristics (e.g., effect of ball size on player reaction and racket acceleration during the tennis volley [59])
- use of technical information to match equipment with athlete, or in crew selection processes (kayaking [260])
- synthesis of athlete motion in competitive game-play by finding related action sequences in a database of reference motions using a sparse set of measurements from the performance (exemplified for tennis shots [177,301])
- differentiation between categories of athletes, based on key performance parameters and technique (elite and sub-elite for ice hockey blade–puck interaction [206,309] and for air pistol shooters [303], and highly trained inter-collegiate and untrained runners in terms of acceleration economy [224])
- monitoring fatigue during running [295]
- determine an optimal gradient and speed during uphill ski mountaineering [254,255].

Wearable sensors have also been embedded into sport-specific feedback and coaching systems which may be used for training and race analysis, providing to the coach/athlete real-time, visual, tactile, and auditory information, as well as data to be analysed immediately. These systems must be designed to comply with user requirements (coaches, biomechanists, and athletes), reliability and sport-specificity being the most important [24]. Acoustic and visual feedback systems have been developed both for sweep rowing [62,275] and sculling [58,116,205,217], for example, performing start and steady-state analysis, under training and race conditions, based on boat velocity and stroke rate [217]. Another promising sport for feedback use is swimming, whose performance and technique parameters, such as CoM velocity, arm strokes, distance per arm stroke, body balance, and body rotation, are assessed and provided to the coach/athlete through a user-friendly interface [24,63,110]. Proof of concept for coaching/training systems has also been demonstrated in skiing [231], golf [147], baseball swing [146], shot-put [139], volleyball [306], and weightlifting [272].

#### 3.4.2. Match Analysis and Load Monitoring/Physical Demands Assessment (61 Papers)

All but one of the studies performed during competition belong to this category (18 papers), while 38 were held in the field during training. Most of the match analysis involving IMUs was carried out using Integrated Technology (IT). IT was developed to increase the sensitivity of GPS systems to abrupt changes in running velocity, direction, and to impacts, typical of most team-sports, by incorporating high-frequency (100 Hz) tri-axial accelerometers in GPS systems. Some systems (e.g., Minimax, Catapult Sports, Melbourne, Australia) also incorporate gyroscopes and magnetometers to improve tackle detection and report the direction of travel, respectively. The MIMUs’ role in the context of match analysis is, therefore, complementary to that of GPS systems (see reviews by [15,16,17,18,19]).

Due to the limitation of GPS systems in tracking movement patterns indoors, research based on IT use covers mainly outdoor sports, including American football [317], Australian rules football [73,74,98,111,137,142,143,178,259,298,299], field hockey [253,319], handball [210], Gaelic football [245], netball [86,99], rugby [101,102,127,128,129,130,131,132,133,134,135,176,208,225,226,297,311,312,316], rugby sevens [296], soccer [66,109,144,152,235,249,277], aerial skiing [175], and tennis [163]. In indoor studies, training load was assessed solely using accelerometer data in badminton [113], basketball [234,274], mixed martial arts [165,182,183], tennis [145], and volleyball [92,136,170].

The match analysis performed in team sports is most often based on the body’s CoM kinematics. This provides information on the amount of the mechanical work associated with the CoM during locomotion and contact-based fatiguing movements generated during training and/or competition [74]. This external component of training load can be used as an activity profile measure (i.e., exercise intensity, body or workload marker). External load is generally determined using IT systems, to measure the distances covered in a variety of locomotor classifications or speed zones. To characterise the intensity of player activity in sports entailing frequent collisions, this approach must be complemented with quantification of the contact demands. Success in such sports is also contingent on the ability to tackle and tolerate physical collisions [48]. Monitoring collisions has implications for injury risk, muscle damage, recovery, and training load manipulations, while the number, type, and magnitude of tackles, as a subset of collisions, are recognised to be crucial to match outcome [102,127,130,131,133,176,208,225,296,316]. The ecological validity of tackle detection depends on the sensor type, algorithm, and sport analysed. An acceleration threshold may not be able to set apart tackles from collisions [133], whereas, both assessing body lean with gyroscopes [143] and developing ad hoc algorithms to identify tackles on acceleration signals [176] were shown to improve ecological validity. This, however, may not transfer across sports [142]. A crucial role can also be attributed to filtering, although this role was only assessed for generic impacts [324], and to the assessment of individual devices, particularly for mechanical damage and signal drift errors, to prevent poor validity [336].

To estimate external load, trunk acceleration has been used, although it was poorly correlated with whole body mechanical loading [240]. The parameter commonly assumed to be proportional to external load is the mean squared instantaneous rate of change in acceleration (i.e., the jerk vector amplitude), termed PlayerLoad™ in some proprietary systems. This external load measure has acceptable within- and between-device reliability [74], moderate to high test–retest reliability, and convergent validity with measures of exercise intensity on an individual basis [67]. Some studies distinguished the jerk contribution to external load along different directions [66,67,98,99,143,249] or its accumulation over time [143]. This parameter varies between sports, training modes, player position/role, etc. [16]. For this reason, methods for quantifying external load should undergo a sport-specific validation, as done, for example, for volleyball [92,170], mixed martial arts [165], and Gaelic Football [245]. External load should also be combined with internal training-load measures (e.g., session rating of perceived exertion) to monitor an individual’s response to the training dose [137,144,175,182,208,259,277,316].

Many objectives were pursued using measures of external load, such as

- quantifying physical and technical demands for a specific sport (aerial skiing [175], American football [317], Australian football [259], mixed martial arts [165,182], rugby [130], soccer [109,249,277])
- assessing the effect of playing position (American football [317], Australian Football [73,137,178], handball [210], netball [86,99], rugby [128,130], rugby sevens [296], soccer [152,277]), playing level (elite and sub elite: Australian Football [73], soccer [109,277]), numerical advantage (soccer [235]), playing time [311], playing standard (Australian Football [98], badminton [113], rugby [129,131]), and age group (tennis [163])
- characterising successful teams (rugby [127,129])
- assessing the effect of match score on activity profile and skill performance (Australian football [298])
- determining how external load measures and skill performance contribute to coaches’ perceptions of performance (Australian football [299]) and how these vary between matches (Australian football [178])
- assessing the contribution of running to external load, through a correlation analysis of distance covered (field hockey [253])
- assessing the number of collisions and repeated high-intensity efforts [128]
- assessing differences between different training conditions (rugby [133]) and with competition demands (Australian Football [73], mixed martial arts [183], netball [86], rugby [134], soccer [277])
- determining the best load assessment according to training mode (rugby [316])
- investigating agility demands of small-sided games (Australian football [111]), and closed drills [60]
- inferring information about internal load (soccer [144])
- assessing the impact of playing experience on internal load, as estimated through the external load [137]
- assessing fatigue, from training sessions (rugby [133]), from consecutive days of match play (tennis [145]), and in real time (soccer [66]).

More sophisticated match analysis may complement physiological demands with additional information about players’ movements within their positional zones, for example, assessing an individual’s tactical behaviour with respect to players with the same role [152].

#### 3.4.3. Motor Capacity Assessment (51 Papers)

Exploring the force–velocity relationship during movements that involve complex inter-muscular coordination in a standardised setting can provide useful information when designing strength training programmes [337,338]. To this aim, motor capacity standardised assessments have been developed to determine either velocity or power during the acceleration and deceleration of a constant mass, as is typical in athletic movements (iso-inertial assessment [50,305]). For athletic profiling, these assessments should comprise both measures of the athlete’s maximal velocity (*explosive performance*, also referred to as ballistic performance [13]) and *maximal strength* (possibly when lifting a set of increasing loads in classical overload exercises). Both types of assessment have been performed using MIMUs that provide instantaneous acceleration (and therefore force) data along with an integrated measurement of velocity to determine muscular power: *explosive performance* was assessed for the lower limbs during jumps [13,83,84,92,93,97,100,117,150,166,167,199,213,218,219,220,233,244,251,257,258,281,286] and for the upper limbs [78,141,151,190,236,310]. Force and power production capacity were quantified during *maximum dynamic strength tests* for the upper limbs [76,97,103,172,186,198,252,261,305] and lower limbs [64,65,76,82,97,172,252,265,289]. As an indirect assessment of force production capacity, static muscular stiffness has also been assessed [256]. The accuracy and reliability of both explosive performance [13,14,83,100] and strength assessments [13,50,305] has been widely acknowledged as being dependent on data collection equipment. The MIMU based methods were not always shown to be the most accurate or reliable, but can be considered acceptable according to the subsequent application, once the level of error associated with a given configuration is considered.

*Explosive performance* has been analysed using MIMUs during various vertical jumping activities, such as squat, countermovement, and rebound jumps, or landings. This data is quantified for the lower limbs in terms of jump height, maximal velocity, and peak power, eventually separating concentric and eccentric contributions. Several studies tested the accuracy, reliability, and validity of MIMUs in providing jump height [13,83,84,92,93,115,117,167,199,213,218,220,233,244,251] or other jump related parameters, such as peak knee flexion and trunk lean during landing [115], take-off velocity, contact and flight time [93,167,220,233,257,258], and force and power [97,100,281]. Upper limb ballistic performance has been analysed in terms of maximum velocity, estimated strength, peak power during explosive bench press [141,151,236], and also upper limb ability to elevate the centre of mass during upper limb jumps [190]. Based on current reliability findings, all of the reviewed explosive performance assessments may be implemented and used to support different training issues: (i) to determine indices of efficiency relevant to sport-specific performance [190]; (ii) to distinguish between athletes’ skill levels [166]; (iii) to select the best training programmes to improve performance [219,286]; (iv) to determine the optimal load for ballistic tasks performed with overloads (such as jump squats [14]); (v) to assess explosive strength in relation to various factors (such as the athlete’s respective sport [141], level of engagement (recreational or professional) [141], gender [141], and effect of weight machine design [78,310]).

The reliability and accuracy of velocity and power measures performed using accelerometers during *maximum dynamic strength assessments* were evaluated, often in comparison with other assessment methods for upper limb muscular power [97,305] and during squat lifts [64,97,150,289]. Reliability and reproducibility were also assessed for repetition and time under tension during leg and bench press exercises [76]. The reproducibility of velocity and power estimates during squat lifts [82,172,289] and bench press exercises [172] have been assessed. A critical role has been attributed to participant variability, especially for the lightest loads [82,172], with velocity and acceleration variability having a combined negative impact on power reproducibility [339]. Nevertheless, MIMUs can be considered, within the known limits of reproducibility and accuracy, as a tool for monitoring strength–power potentiating complexes, performed with overloads to describe the load–power and load–velocity relationships for specific exercises [172], to determine the optimal loads for training [14,103,261], to monitor the effects of power training [65,265], and to assess the effect of stability conditions on power production [186].

In this respect, performance related parameters, obtained using sport-specific capacity tests, or during standardised *in-field tests* can be of great benefit. However, thus far, few attempts have been made to develop sport-specific capacity tests measuring the athlete’s performance potential in combat sports [269] or to adapt traditional tests, such as vertical jumps, to a specific sport, as done for soccer for analysing vertical jumps with intention to head [257]. Instrumented in-field tests have been developed mainly to assess the performance of wheelchair users [71,216,250,282,292,307], probably because of the higher quality of signal obtainable by attaching the sensors to the wheelchair or athletes’ upper limbs, thus limiting the effect of soft tissue wobbling. These papers testify the potential of MIMUs to assess agility (repeated turn tests), by quantifying shoulder and elbow joint kinematics [292] or wheelchair kinematics [307], and muscular power (sprint tests) or endurance (distance tests), by quantifying average and peak wheelchair kinematic parameters [71,216,250,282,292]. A reliable assessment of agility drills in match-like conditions enables prospective research during real matches [60,307], as utilised in wheelchair basketball [308]. The description of individual wheelchair mobility can also be used for classification within the sport [308].

#### 3.4.4. Activity Classification (19 Papers)

Activity classification papers cover

- the identification of different movement patterns through simple visual inspection (cross-country skiing [215])
- the identification/evaluation of techniques and styles/tricks [154,164,268], specific movement phases [114], and events (player tackles and collisions during rugby matches [176], putt detection and characterization for golf training progress [171], arm action and ball release performed by cricket fast bowlers during training and competition [227], eventually used for the detection of illegal events [195,322])
- automatic detection and classification of sports activities during wheelchair rugby matches [94] and team sport activities [326]
- automatic classification of training backgrounds and experience levels in runners [184].

Activity classification has been performed by measuring body segments in relative or absolute orientation, to detect sprinting, jogging, walking [52,195,232,326], jumping, cutting, kicking [52], dribbling, ball strike, tackling [232], swimming style [164,304], trampoline routines [161], climbing phases [114], rugby collisions [176], volleyball actions [306], tennis strokes [301], golf putting [171], and aerial manoeuvres in half-pipe snowboarding [159] and skateboarding [154].

Automatic classification (i.e., mapping input data to a predefined category) may be carried out through the following procedure: (i) acquiring an annotated dataset in which, in cases of supervised classification, each datum is associated to a class label; (ii) pre-processing data by filtering, down-sampling or windowing to identify data segments to be classified; (iii) extracting discriminative features, i.e., quantities that hold the information to be used for classification; (iv) training the selected classifier using the training set; (v) using the generated classification rules for prediction of the test set. Activity classification solutions are then often cross-validated, by running several different splits between the training and test sets, to better assess the quality of the methods. Common approaches for this are the N-fold cross validation and the leave-one-subject-out cross-validation method. The selected features/patterns should encode important and intuitive properties of the athlete’s body configuration while remaining constant under global variations. It is desirable that stronger correlations exist between similar features/patterns and poorer correlations between dissimilar features/patterns. They can be simple time/frequency features, such as means, standard deviations, entropy, dominant frequencies, or can be extracted using Discrete Wavelet Transform. Unsupervised approaches for classification cluster the extracted features, often based on k-means analysis, with a predefined cluster number or by selecting the cluster number within a consensus clustering framework [278]. Supervised approaches train the classification system using a set of data with a known classification. The classifiers of this kind used in the revised literature are decision trees (random forest training algorithms [52,326], logistic model trees [232,326]); stepwise discriminant analysis of principal components [184]; k-Nearest Neighbour algorithms (IBk [232], Lazy IBk [52]); Artificial Neural Networks (RBF Network [52], MLP network [232]); Support Vector Machines [176,232,326]; Naïve Bayesian classifiers [52,154,232]; fractals [94]; Hidden Markov or Semi-Markov models [171,304]; and Hidden Conditional Random Field [176].

### 3.5. Device Characteristics

A systematic analysis of sensors’ positioning and configuration, as implemented in the reviewed studies, is provided in Table 5 and Table 6; sensors attached on the athlete’s body segments are reported in Table 5, while those attached or inserted into sport equipment are depicted in Table 6.

In regard to the type of inertial sensors used, more than half of the reviewed studies (57%) exploited only 1D/3D accelerometers. In the remaining cases, the measurement of the accelerometer was integrated with other sensors, namely, gyroscopes (25%) or gyroscopes and magnetometers (15%). Only a few studies used 1D/3D gyroscopes in isolation (3%). In regard to the sensors’ features, the ranges of the accelerometers were largely below 10 g (71%) and more than half of the gyroscopes had a range below 1200 deg/s (61%). The most used commercial system was the MinimaxX (considering all the different versions produced by Catapult) followed by Myotest (Myotest), SPI-PRO (GPSports), Xsens systems (Moven suit and MTx sensor), and Physilog (GaitUP). On the other hand, 68% of the reviewed studies implemented custom-made devices and 15% reported no information in this respect.

With regard to IMU number and position, 67% of the studies exploited a single sensor configuration, 24% used two to five sensors, 7% used more than five sensors, and 2% did not report this information. Typically, the single sensor configuration was inserted into the sport equipment or attached to the trunk–pelvis segment or distal segments, such as the hand and foot. Considering the sports most frequently analysed—swimming and running—the single sensor configuration was preferred in 69% and 60% of the studies, respectively. Several procedures were adopted to attach the sensor onto the athlete’s body segments, from double-side tapes, elastic stretch bands, straps, and Velcro fasteners, to body suit and customized vests (Figure 3). When the sensor was attached to the sport equipment, glue was typically used.

## 4. Discussion

### 4.1. General Trends

Coaches, athletes, and support staff are benefitting more and more from the use of magneto-inertial sensor technology. In recent years, a high number of publications have testified that this technology is now suitable for assessing player capacity, technique, and match workload, both for individual athletes and sports teams (Figure 2). Monitoring these aspects can enhance training design and evidence biomechanical fatigue, thus facilitating the development of appropriate injury reduction interventions [3,340].

*Technique analysis* has been performed for more than 37 sports using MIMUs, to obtain predominantly spatio–temporal parameters, centre of mass kinematics, and body segment orientation, spanning a broad spectrum of objectives. The majority of this literature focuses on describing technique, rather than developing tools to correct technique because exploitation of inertial sensors in sport performance evaluation is rather recent. Only after gaining a deep understanding of MIMUs patterns, for different athletes’ levels, it will be possible to use this tool to revise or to improve the technique of a given athlete. Regarding experimental errors, gyroscopes have demonstrated greater limitations (i.e., drift) than accelerometers, but they are essential when obtaining orientation both as a parameter in dynamic conditions and when removing gravity from acceleration data. As a result, orientation and joint angle estimates can be inaccurate; accuracy depends on task duration, intensity, and soft tissue wobbling, which are dictated by the task and body segment analysed. Drift errors also hinder the accuracy of velocity and position estimates, as obtained through the numerical integration of MIMU signals.

Combined IMU and GPS data have been used to obtain sensitive and holistic measures of *external loading* in team sports. Specifically, they enable the detection of abrupt changes in running velocity and direction, as well as impact identification and classification [67]. Data have also been used to quantify the technical demands of team sports and have proved to be sensitive to the effect of factors influencing success. These data can support the manipulation of team sport parameters (e.g., rules, field size, player numbers and position, coach feedback, etc.), which facilitates optimisation of training stimuli [48,182,341].

MIMU-derived information has proven to be adequate in assisting routine *capacity assessment* along the vertical direction during explosive performance and maximum dynamic strength tests. This supports the evaluation of learning and progression/regression processes and, therefore, enhances the efficacy of a planned training schedule and the compilation of a training diary. The accuracy and reliability of these assessments are dependent on data collection procedures and equipment. This currently inhibits the development of more complex instrumented in-field tests, occurring in the horizontal plane (e.g., Yo-Yo test, Reactive Agility test).

The current decade has seen a rapid increase in the availability and use of wearable technology [342,343,344,345]. As sensor utilisation rises across sports and monitoring is allowed during competition (e.g., rugby, baseball, tennis) [346], the challenge becomes providing more intelligent, real-time, accurate information, making it user friendly and offering coaches and athletes actionable insights [347,348]. In fact, amongst coaches, there is a perceived lack of MIMU related knowledge and known low usage rates, according to a survey of 500 swimming coaches [349]. This aspect can be explained by the fact that most signals obtained from MIMUs are less intuitive than, for example, the position of an anatomical landmark that can be easily investigated and interpreted using standard video analysis. Technology usage is hindered by the expense of adoption and implementation, by the skills required for data collection, extraction, and interpretation (indoor team sports [350]), as well as by the current lack of standardisation procedures [351]. On one hand, technicians and engineers should work on the development of methods aimed at providing coaches with metrics that are informative, concise, and easy to interpret. On the other hand, coaches, athletes, and support staff could enhance their likelihood of use and understanding by partaking in the development process of second generation monitoring systems, by identifying training needs and opportunities [350,352]. Further, standardisation of relevant procedures is necessary to maximise the technology’s potential.

### 4.2. Technological Advancements and Future Developments

Technological advancements may cause significant changes with lasting impacts on the performance and spectatorism of a sport [159,353]. The most significant recent developments have led to increased commercial sensor affordability and availability of sensor fusion technology and improved power management strategies (for example turning off a gyroscope when not necessary) [347]. Sampling frequency and full range scales are now adequate for most sport applications. The technology has reached a stage whereby, for mainstream sports, data collection is easily possible and affordable. For example, new generation gyroscopes allow tracking of fast rotational movements thus enabling the development of in-field training or monitoring devices (tennis [54]), and support tools for referees [354]. The cost of MEMS has been driven down by smartphones which have built-in sensors, including accelerometers, GPS, and often gyroscopes [347]. This enables smartphones to provide information on the motion, environment, position, and orientation of a device to both inexperienced users and experts alike. However, there is limited evidence supporting the use of smartphones over MIMUs [232,355,356]. This is potentially due to wearability issues [357] and inaccurate sensors. Wearability has been improved by using smaller sensors which transmit data back to the smartphone; alternatively, MIMUs are being substituted by smart fabrics which use garments as a wearable platform in which to embed sensors and processing units and appear as a potential future alternative/integration to MIMU [358]. To partially overcome the accuracy issue, MIMUs can be complemented by new sensors within a sensor fusion framework; ultra-wideband ranging sensors promise to estimate both orientation and position [359], whereas barometer sensors estimate only position [360,361].

Until recently, only professional teams and elite individual athletes had access to highly specialised technology. Recreational athletes were restricted to the use of low cost IMUs, such as step counters, whose outcome is often dissatisfactory. However, increasingly more products are being designed for broad participation; often products designed for elite use are later tailored to recreational athletes (examples with costs ranging $70–200 can be found in Lightman [347]). Consequently, it is expected that recreational athletes develop a desire for more detailed and accurate performance data and an increased demand for affordable higher quality intensity/performance trackers, at an intermediate cost (wearables 2.0, potentially including virtual reality). This market demand is driving the democratisation of wearable technology. In the near future, fitness trackers requiring manual and subjective interpretation will be replaced by machine learning tools able to provide real-time monitoring and automatic and objective analysis. Furthermore, this data will provide clear actionable insights, benefitting users of all levels.

To support athletes in adapting their training and technique with useful insights, feedback systems are required [362]. These systems also reinforce the relationship between the coach’s observations and the athlete’s subjective perception of force and movement. The number and type of sensors is of paramount importance to their efficiency and success [363]. Further, visual feedback can help people with limited experience in MIMU use interpret the signal patterns [4]. Communication technologies allow athletes to document and share their training data, which may enable remote training supervision [357,364].

For these insights to become actionable, coaching systems must integrate sensor measurements with machine learning systems for the purpose of providing intelligent, online feedback. This enables tactical use and workload management, in addition to post game analysis [357,365]. Physical movement monitoring first requires the definition of a linguistic framework to represent the data symbolically [366]. Real-time or near real-time systems based on robust wireless network solutions can provide quick access to information. Then, the detection of key information and event classification can be performed using models for pattern recognition and classification of the motion or activity performed. Different methods and models, such as Neural Networks, Hidden Markov models, and Support Vector Machines, have proven to be appropriate for this purpose (see *Activity classification* for further examples). This approach is essential for feedback systems aiming to recognise and evaluate the quality of specific techniques in the field [301], and eventually during technical–tactical sessions [306]. Machine learning can also automatically provide feedback on how to continue exercising and/or adjust smart sports equipment, depending on environmental conditions [367]. We hypothesise that, using a similar approach, athlete capacity assessment can also be further improved to include measures of sprint ability and agility and by assessing sport-specific tasks covering the entire force–velocity–power spectrum.

### 4.3. Guidelines and Standards

There is a call for the standardisation of data collection and analysis procedures amongst several contexts. For team sports, standardisation of physical demands and external load quantification are required, to facilitate comparison between skill levels and within a specific sport [101]. For capacity assessment, standardisation of power measurements is required to further understand the load–power relationship and the training adaptation mechanisms [50,368]. A list of general key guidelines is here reported that may help in assessing sensor quality, performing sensor calibration and anatomical calibration, properly fixing the sensors and processing the data (Table 7). Nevertheless, complete guidelines and standards for testing should be application- and sport-specific since, for a selected parameter, error is dependent on the task. Additionally, error compensation methods do not necessarily transfer across sports [143]. Therefore, the choice of methodology should be driven by the parameters and the task, with a balance between accuracy and practicability as well as consideration of environmental factors. Parameter-specific recommendations for both sensor type and sensor placement have been developed for running [243], but more research is required that is specific to other sports and parameters. In open skill sports, for example, the intrinsic variability must be considered in order to obtain reliable performance indicators [369]. Therefore, the sport scientist must have in-depth knowledge of the technology in order to make an informed decision, eventually combining different levels of sensor accuracy according to the aims [370].

## 5. Conclusions

This review demonstrates that magneto- inertial technology, continuously gaining momentum in sports biomechanics, is a reliable tool able to benefit athletes of all levels, especially when complemented within a sensor fusion network. This can ultimately extend and enhance athletes’ careers through improved injury prevention and training specificity. While this reliability is fully exploited in the laboratory, there must be a compromise between the technology’s potential and practicality in the field. When this compromise is not reached, mainly due to the lack of proper standardisation of data acquisition and of tools for the subsequent analysis, the technology may suffer from low usage rates amongst coaches. Better collaboration between the sport biomechanist and practitioner would facilitate overcoming this issue. Future research should focus on ease of use and improved error compensation and analysis procedures able to provide coaches with informative, concise, and easy to interpret metrics. In doing so, MIMUs can facilitate the provision of actionable insights for both the athlete and coach.

## Figures and Tables

**Table 1:** Inclusion and exclusion criteria considered for the current systematic review.

Criteria	Definition
Measurement instruments	magneto-inertial sensors
Motor tasks	tasks of interest in a sport context
Type of assessment	included: motor capacity performance related parameters intensity measures for match analysis refereeing parameters excluded: oxygen uptake, metabolic cost, and activity monitoring parameters exclusively related with the risk of injury
Publication type	journal papers
Cohort under investigation	recreational, experienced, and elite athletes

**Table 2:** Boolean search strategy.

Database Keywords
**Web of Science:** (“accelerometry” OR “accelerometer” OR “gyroscope” OR “inertial sensor” OR “inertial measurement unit” OR “wearable sensor” OR “wearable system” OR “wearable device” OR “IMU” OR “MEMS”) AND (sport OR “baseball” OR “basketball” OR bicycling OR “boxing” OR “football” OR “golf” OR “gymnastics” OR “hockey” OR “martial arts” OR “tai ji” OR “karate” OR “taekwondo” OR “mountaineering” OR “racquet sports” OR “tennis” OR “cricket” OR “softball” OR “badminton” OR “running” OR “jogging” OR skating OR “snow sports” OR ski * OR “soccer” OR “snowboard” OR swimming OR “diving” OR “track and field” OR “volleyball” OR “weight lifting” OR “wrestling”) AND (humans OR athletes) NOT (patients OR pathology OR animals OR “physical activity” OR “energy expenditure”) limit to English
**Scopus:** “accelerometry” OR “accelerometer” OR “gyroscope” OR “inertial sensor” OR “inertial measurement unit” OR “wearable sensor” OR “wearable system” OR “wearable device” OR “IMU” OR “MEMS” AND sport AND humans OR athletes AND NOT patients OR pathology OR animals OR “physical activity” OR “energy expenditure” limit to English
**Pubmed:** (“accelerometry” OR “accelerometer” OR “gyroscope” OR “inertial sensor” OR “inertial measurement unit” OR “wearable sensor” OR “wearable system” OR “wearable device” OR “IMU” OR “MEMS”) AND (sports [Mesh] OR karate OR taekwondo OR “cricket” OR softball OR badminton) AND (humans [Mesh] OR athletes [Mesh]) NOT (patients OR pathology [Mesh] OR animals OR “physical activity” OR “energy expenditure”) AND “English” [Language]
**Sport Discus:** (“accelerometry” OR “accelerometer” OR “gyroscope” OR “inertial sensor” OR “inertial measurement unit” OR “wearable sensor” OR “wearable system” OR “wearable device” OR “IMU” OR “MEMS”) AND (sport OR “baseball” OR “basketball” OR bicycling OR “boxing” OR “football” OR “golf” OR “gymnastics” OR “hockey” OR “martial arts” OR “tai ji” OR “karate” OR “taekwondo” OR “mountaineering” OR “racquet sports” OR “tennis” OR “cricket” OR “softball” OR “badminton” OR “running” OR “jogging” OR skating OR “snow sports” OR ski * OR “soccer” OR “snowboard” OR swimming OR “diving” OR “track and field” OR “volleyball” OR “weight lifting” OR “wrestling”) AND (humans OR athletes) NOT (patients OR pathology OR animals OR “physical activity” OR “energy expenditure”) AND (LA (English))

* Entails truncation, i.e., finding all terms that begin with the string of text written before it.

**Table 3:** Number of papers in which each sport has been analysed using sensors. Multiple counts are allowed for papers performing comparative studies across sports.

Team Sports		Other Individual Sports		Cyclic Sports		Winter and Outdoors Sports	
rugby	20	tennis	7	distance running	46	alpine skiing	7
Australian football	12	golf	5	swimming	34	climbing	5
soccer	10	weightlifting	4	cycling	10	cross-country skiing	4
baseball	5	mixed martial arts	3	rowing	8	ski jumping	4
cricket	4	boxing	2	sprint running	3	snowboarding	4
field hockey	4	diving	2	kayaking	2	ski mountaineering	3
volleyball	4	javelin throw/shot-put	2	race walking	1	aerial skiing	1
ice hockey	3	karate	2			roller skiing	1
netball	3	artistic gymnastics	1	**Motor capacity**		skateboarding	1
basketball	2	badminton	1				
wheelchair basketball	2	bowling	1	jumping	24		
American football	1	fencing	1	overload training	20		
contact sports	1	horse riding	1	agility tests	7		
Gaelic football	1	shooting	1				
handball	1	taekwondo	1				
softball	1						

**Table 4:** Type of parameters, assessments and sports analysed to support sport technique analysis.

Parameter Type			Type of Assessment	Sport
Spatio-temporal	temporal	non-cyclic tasks	measure critical temporal events	blade–puck contact time in ice hockey
				cricket ball bowling and release
			detect task phases and critical events	artistic gymnastics springboard jumps
				baseball swing
				bowling
				cricket bowling
				diving trampoline jumps
				golf
				half-pipe snowboard
				instep kick
				javelin throw
				karate front kick
				ski jumping
				soccer turning manoeuvres
				swimming tumble turn and start
		cyclic tasks	characterize cyclic stride/step/stroke event	cricket ball delivery
				kayaking
				multi-person rowing
				running
				single sculler rowing
				skating
				swimming
			revolution rate	bowling ball
				cycling
			detect task phases	cross-country skiing
				front crawl
				ice hockey skating
				running on a track
				running on a treadmill
				sprint running
				uphill mountaineering
	spatial		Step/cycle length	swimming
				treadmill running
**Centre of mass (CoM)**	velocity	instantaneous		rowing
				alpine skiing
				cycling
				running
				simulated cross-country skiing
				snowboarding
				swimming
				vertical jumping
		average		cross-country skiing
				running
				running cycle
				swimming cycle
				swimming lane
				swimming turning
				uphill mountaineering
	displacement	forward		alpine skiing
				cross-country skiing
				jumps while skiing, snowboarding, mountain biking
				running
				ski jumping
				snowboarding
				uphill mountaineering
		vertical		running
				golf swing
				ski jumping
	acceleration		event detection and amplitude	running impacts
				landing from horizontal jump
				tennis racket shock
			variations in acceleration amplitude	running performance, economy, symmetry
**Objects**	orientation	whole body movements	ski horizontal and V-opening angles	ski jumping
			ski inclination	alpine skiing
			boat–oar stroke angle	rowing
			boat orientation	
			bicycle roll and crank angle	cycling
		swing	club face orientation	golf
			bat orientation	baseball swing
	kinematics	whole body movements	springboard kinematics	gymnastic vaulting
			boat kinematics	rowing
			oar acceleration	
			seat position	
			road incline	running
			shot acceleration	shot put
			ski velocity	ski jumping
		swing	bat position and linear velocity	baseball swing
			club head kinematics	golf
			racquet head forward velocity	tennis
		overarm throw	ball velocity at release	baseball swing
				softball
			javelin kinematics	javelin throw
		sidearm/underarm movements	ball velocity	bowling
				cricket bowling
			foil speed	fencing lounge and *touche*
			puck velocity	ice hockey
Body segments	orientation		trunk rotation	aerial manoeuvres in half-pipe snowboard
				bike riding
				swimming
			trunk inclination	golf swing
				running on a track
				snowboarding
				sprint start
			lower limb orientation	alpine skiing
				ski jumping
				trampoline jumps
			upper limb orientation	baseball pitching
				running on a track
				tennis
			pelvis orientation	climbing
				ski jumping
				swimming
			gravity vector removal	running
				swimming
				various tasks
	kinematics		postural tremor	air pistol shooting
			lower limb vibrations	off-road and road cycling
			take off velocity and angle	standing horizontal jump
			shoulder, elbow, wrist displacement	baseball pitching
			heel lift and forearm acceleration	running
			tibial linear acceleration	depth jumps
			tibial angular and linear acceleration	instep kick
			tibial angular and linear velocity	
			pelvis jerk	climbing
	joint kinematics	wrist	angular displacement	swimming
				tennis
		shoulder	angular displacement	running
				swimming
				tennis
		elbow	angular displacement	swimming
		hip	angular displacement	cycling
				horse riding
				karate front kick
			angular velocity	karate front kick
		knee	angular displacement	alpine skiing
				ski jumping
				snowboarding
			angular velocity	karate front kick
				running
		ankle	angular displacement	cycling
				ski jumping
				soccer specific tasks
		main joints	angular displacement	trampoline jumps
Dynamics	dynamics	external forces	aerodynamic force	ski jumping stable flight
			dissipative forces	bowling ball
				ski racing
			ground reaction forces	ski jumping take-off
				ski racing
				springboard during diving
			impact force	straight punch
			power	ski racing
		joint moments	elbow	baseball pitching
			lower limb	instep kick
				snowboarding turns

**Table 5:** Sensors’ positions and configurations on the athlete’s body (front and back views). The different positions are coded with specific body segment capital letters (C—chest-back; U—upper arm; A—forearm; W—wrist; H—hand; P—pelvis; T—thigh; S—shank; F—foot) and numbers (to distinguish different positions for the same body segment). The different configurations are specified in terms of type of device (accelerometer, gyroscope, magnetometer) and number of dimensions (one-, two-, three-dimensional). Each configuration is reported in a different column.

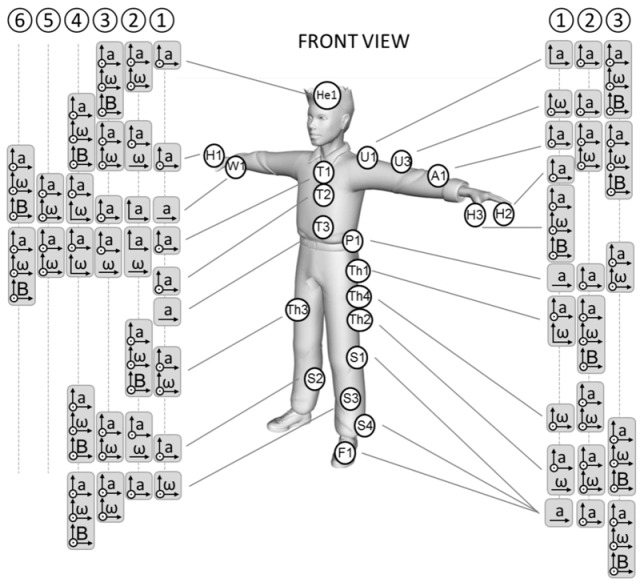						
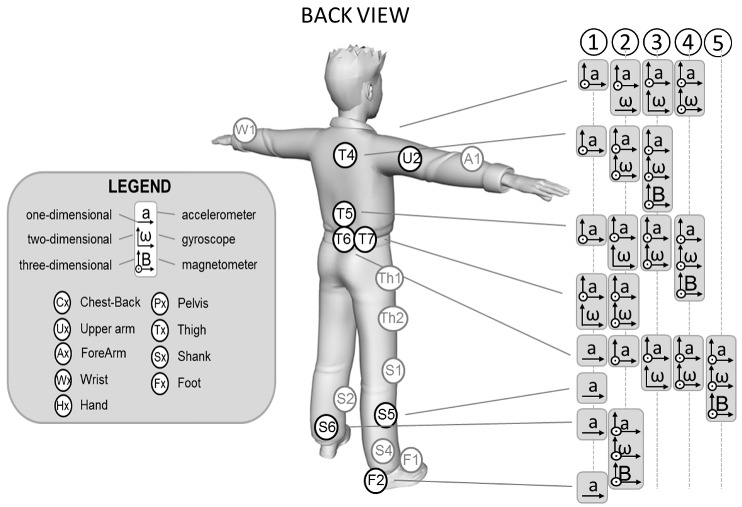						
**Device Position**	**1**	**2**	**3**	**4**	**5**	**6**
He1 (head-helmet)	[68]	[122]	[212,302]			
U1 (acromion)	[58]	[303]	[119]			
U2 (upper arm posterior)	[80]	[53,54]	[147]	[168,185,267,295,322]		
U3 (upper arm)	[331]	[269]				
A1 (fore arm)	[177,247,303]	[64,107,108,158,185,322]	[114,119,161,211,212,278]			
W1 (wrist)	[71,323]	[76,295]	[63,80,156,164,261,269,288,332]	[146,147]	[266]	[76,295]
H1 (third metacarpal bone)	[303]	[53,158]	[301]	[119]		
H2 (index finger)	[303]					
H3 (thumb)	[180]					
T1 (sternum, clavicles)	[112]	[115]	[53,54]	[146]	[122]	
T2 (sternum bottom)	[174,177,274,306]					
T3 (navel)	[166]					
T4 (upper back, T6–T10)	[56,60,63,66,67,73,74,80,86,98,99,101,102,111,130,131,132,135,137,143,152,163,165,175,183,208,225,226,231,232,234,235,240,241,242,249,253,259,274,277,296,297,311,312,317,319]	[128,129,134,136,182,295,326,330,331]	[161,178,210,215,227,266,295,333]			
T5 (L1–L3)	[63,84,174,177,184,191,197,223,224,320,321]	[24]	[69,70,192,193,290]	[119,212]		
T6 (L4, L5, sacrum, or lower back)	[155]	[61,63,67,72,80,85,110,113,162,170,190,194,196,200,201,203,213,234,237,244,273,304,332]	[147,285]	[41,88,89,90,104,105,106,107,122,168,220,248,251,257,262,263,291]	[114,199,211,238,258,278,281]	
T7 (5 cm left of L5)	[146]	[159]				
P1 (iliac crest)	[264]	[83,218,219,264,265]	[52]			
Th1 (great trochanter-hip)	[147]	[280]				
Th2 (distal thigh)	[126]	[88,89,90,115,273]	[96]			
Th3 (frontal thigh)	[52,91,231]	[214,228,238,294]				
Th4 (mid lateral thigh)	[331]	[122]				
S1 (head of fibula)	[117,157,229,230,313]	[248,295]				
S2 (medial shank)	[243]	[62,124,125,126]	[52,88,89,90,91,115,231,256,273,328]	[55,161,211,238]		
S3 (frontal shank)	[140,222,228]	[177,240]	[91,122,168]	[214,294]		
S4 (ankle)	[148,248,283,294,295]	[269]	[167]			
S5 (lateral shank)	[256]					
S6 (lower postural shank)	[256]	[212]				
F1 (shoelace)	[160,209,318]	[87,160,202,209,294,318]	[55,57,114,214,278,295]			
F2 (heel)	[118,148,162]					
Moven suit (Xsens Technologies, The Netherlands) (He, T2, U3, A1, H1, Th2/Th4, S5/S6, F1, T6)	[43,75,138,161,188,189,292,300]					

**Table 6:** Sensors’ positions and configurations attached or inserted into the sport equipment. The different positions are coded with specific equipment capital letters (B—barbell; St—strike; L—locomotion) and numbers (to distinguish different positions for the same type of equipment). The different configurations were specified in terms of type of device (accelerometer, gyroscope, magnetometer) and number of dimensions (one-, two-, three-dimensional). Each configuration is reported in a different column.

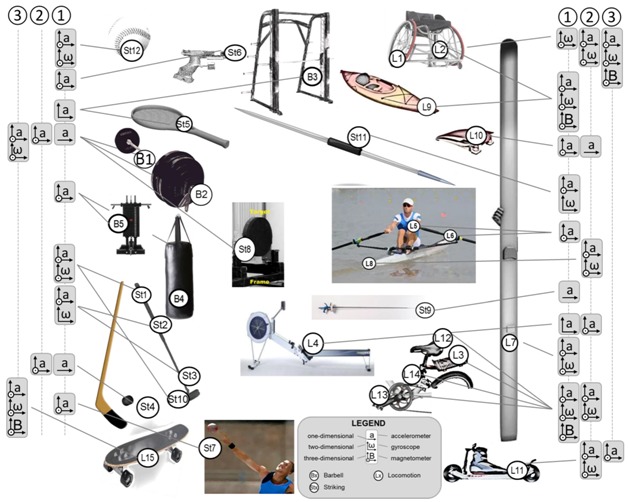			
**Device position**	**1**	**2**	**3**
B1 (barbell close to handle)	[186]	[65,82,100,123,198,272,310]	[78,289]
B2 (barbell extremity)	[172]	[150,151,271]	
B3 (on the smith press)		[305]	
B4 (punching bag)	[79]		
B5 (weight stack)	[252,284]		
St1 (golf shaft)	[181]		
St2 (golf lower hand)	[147]		
St3 (golf near head)	[147]		
St4 (hockey puck)	[206]	[309]	
St5 (racquet)	[59]		
St6 (pistol)	[303]		
St7 (on the shot put)	[139]		
St8 (frame and target)	[239]		
St9 (on the foil)		[329]	
St10 (golf on the club head)	[171]		
St11 (javelin)	[270]		
St12 (baseball/softball)	[221]		
L1 (wheelchair wheel)	[94,216,282]	[250,308]	
L2 (wheelchair frame)		[308]	[71,282,307]
L3 (rear bike)	[302]	[330]	
L4 (rowing seat)	[58]		
L5 (rowing oar)	[205,295]		
L6 (rowing boat)	[116,205]	[260]	
L7 (skis)	[88,90,121,231,254,255]		
L8 (top of the boat)		[217,275,287]	
L9 (on the kayak)		[169]	
L10 (on the springboard)	[187]	[173]	
L11 (roller ski/skate chassis)		[268]	[293]
L12 (bike seat)			[95,212]
L13 (bike crank)			[95]
L14 (bike frame)		[331]	

**Table 7:** Guidelines for Magnetic and Inertial Measurement Unit quality assessment, calibration, fixing and data processing.

Guidelines for Magnetic and Inertial Measurement Unit Use
**Quality Assessment** 1. avoid ferromagnetic disturbances when possible (no iron or magnetic fields) 2. assess accuracy following specific guidelines for sensor spot checks (e.g., [371,372])
**Calibration** 3. re-calibrate accelerometers, gyroscopes [373,374,375], and magnetic sensors [376,377] to improve poor accuracy before each acquisition session when possible 4. carefully perform anatomical calibration, either functional or point-based, especially when assessing joint kinematics [38]
**Fixing** 5. avoid restricting the range of movement 6. limit the movement between body and device (avoid tape) 7. for tasks entailing impacts, avoid elastic belts for fixing 8. avoid areas with “wobbling” soft tissues (fat or muscles) and areas close to joints
**Data Processing** 9. low-pass filter with various cut-off frequencies depending on the analysed sport task [25,378] 10. use ad hoc algorithms to compensate for drift errors [21,371] 11. compensate ferromagnetic disturbances indoor [334,379] 12. refer to appropriate validation based on reference data or literature that is sport and task-specific 13. interpret data within the limits set by the quality assessment

## Supplementary Materials

A database with the details of all papers that are not reviews and were included in the systematic review is available online at http://www.mdpi.com/1424-8220/18/3/873/s1.

## References

[1] Chambers R., Gabbett T.J., Cole M.H., Beard A. (2015). The use of wearable microsensors to quantify sport-specific movements. Sports Med..

[2] Hood S., McBain T., Portas M., Spears I. (2012). Measurement in sports biomechanics. Meas. Control.

[3] Li R.T., Kling S.R., Salata M.J., Cupp S.A., Sheehan J., Voos J.E. (2016). Wearable performance devices in sports medicine. Sports Health.

[4] Lee J.B., Ohgi Y., James D.A. (2012). Sensor fusion: Let’s enhance the performance of performance enhancement. Procedia Eng..

[5] Mendes J.J.A., Vieira M.E.M., Pires M.B., Stevan S.L. (2016). Sensor fusion and smart sensor in sports and biomedical applications. Sensors.

[6] Baca A. (2017). Innovative diagnostic methods in elite sport. Int. J. Perform. Anal. Sport.

[7] Knight J.F., Bristow H.W., Anastopoulou S., Baber C., Schwirtz A., Arvanitis T.N. (2007). Uses of accelerometer data collected from a wearable system. Pers. Ubiquitous Comput..

[8] Wagner J.F. (2018). About Motion Measurement in Sports Based on Gyroscopes and Accelerometers—An Engineering Point of View. Gyroscopy Navig..

[9] De Magalhaes F.A., Vannozzi G., Gatta G., Fantozzi S. (2015). Wearable inertial sensors in swimming motion analysis: A systematic review. J. Sports Sci..

[10] Callaway A., Cobb J., Jones I. (2009). A comparison of video and accelerometer based approaches applied to performance monitoring in swimming. Int. J. Sports Sci. Coach..

[11] Higginson B.K. (2009). Methods of running gait analysis. Curr. Sports Med. Rep..

[12] Norris M., Anderson R., Kenny I.C. (2014). Method analysis of accelerometers and gyroscopes in running gait: A systematic review. J. Sports Eng. Technol..

[13] McMaster D.T., Gill N., Cronin J., McGuigan M. (2014). A brief review of strength and ballistic assessment methodologies in sport. Sports Med..

[14] Dugan E.L., Doyle T.L.A., Humphries B., Hasson C.J., Newton R.U. (2004). Determining the optimal load for jump squats: A review of methods and calculations. J. Strength Cond. Res..

[15] Aughey R.J. (2011). Applications of GPS technologies to field sports. Int. J. Sports Physiol. Perform..

[16] Cummins C., Orr R., O’Connor H., West C. (2013). Global positioning systems (GPS) and microtechnology sensors in team sports: A systematic review. Sports Med..

[17] Dellaserra C.L., Gao Y., Ransdell L. (2014). Use of integrated technology in team sports: A review of opportunities, challenges, and future directions for athletes. J. Strength Cond. Res..

[18] Scott M.T.U., Scott T.J., Kelly V.G. (2016). The validity and reliability of global positioning systems in team sport. J. Strength Cond. Res..

[19] Waegli A., Skaloud J. (2009). Optimization of two GPS/MEMS-IMU integration strategies with application to sports. GPS Solut..

[20] World Health Organization (2001). International Classification of Functioning, Disability and Health (ICF).

[21] Sabatini A.M. (2011). Estimating three-dimensional orientation of human body parts by inertial/magnetic sensing. Sensors.

[22] Luinge H.J., Veltink P.H. (2005). Measuring orientation of human body segments using miniature gyroscopes and accelerometers. Med. Biol. Eng. Comput..

[23] Sabatini A.M. (2005). Quaternion-based strap-down integration method for applications of inertial sensing to gait analysis. Med. Biol. Eng. Comput..

[24] Le Sage T., Bindel A., Conway P.P., Justham L.M., Slawson S.E., West A.A. (2011). Embedded programming and real-time signal processing of swimming strokes. Sports Eng..

[25] Hamill J., Caldwell G.E., Derrick T.R. (1997). Reconstructing digital signals using Shannon’s sampling theorem. J. Appl. Biomech..

[26] Woodman O. (2007). An Introduction to Inertial Navigation.

[27] Camomilla V., Dumas R., Cappozzo A. (2017). Human movement analysis: The soft tissue artefact issue. J. Biomech..

[28] Liu T., Inoue Y., Shibata K. (2009). Measurement of soft tissue deformation to improve the accuracy of a body-mounted motion sensor. J. Med. Devices.

[29] Lafortune M.A. (1991). Three-dimensional acceleration of the tibia during walking and running. J. Biomech..

[30] Cereatti A., Bonci T., Akbarshahi M., Aminian K., Barré A., Begon M., Benoit D.L., Charbonnier C., Dal Maso F., Fantozzi S. (2017). Standardization proposal of soft tissue artefact description for data sharing in human motion measurements. J. Biomech..

[31] Nokes L., Fairclough J.A., Mintowt-Czyz W.J., Mackie I., Williams J. (1984). Vibration analysis of human tibia: The effect of soft tissue on the output from skin-mounted accelerometers. J. Biomed. Eng..

[32] Forner-Cordero A., Mateu-Arce M., Forner-Cordero I., Alcántara E., Moreno J.C., Pons J.L. (2008). Study of the motion artefacts of skin-mounted inertial sensors under different attachment conditions. Physiol. Meas..

[33] Decker C., Prasad N., Kawchuk G.N. (2011). The reproducibility of signals from skin-mounted accelerometers following removal and replacement. Gait Posture.

[34] Bachmann E., Yun X., Peterson C. An investigation of the effects of magnetic variations on inertial/magnetic orientation sensors. Proceedings of the IEEE International Conference on Robotics and Automation.

[35] Kalman R. (1960). A new approach to linear filtering and prediction problems. J. Basic Eng..

[36] Madgwick S.O.H., Harrison A.J.L., Vaidyanathan A. Estimation of IMU and MARG orientation using a gradient descent algorithm. Proceedings of the IEEE International Conference on Rehabilitation Robotics.

[37] Mahony R., Hamel T., Pflimlin J. (2008). Nonlinear complementary filters on the special orthogonal group. IEEE Trans. Autom. Control.

[38] Cereatti A., Della Croce U., Sabatini A.M., Müller B., Wolf S.I. (2017). Three-dimensional human kinematics estimation using magneto-inertial measurement units. Hand Book of Human Motion.

[39] Walker J.A. (1998). Estimating velocities and accelerations of animal locomotion: A simulation experiment comparing numerical differentiation algorithms. J. Exp. Biol..

[40] Yuan Q., Chen I. (2014). Localization and velocity tracking of human via 3 IMU sensors. Sens. Actuators A Phys..

[41] Myklebust H., Gløersen Ø., Hallén J. (2015). Validity of ski skating center-of-mass displacement measured by a single inertial measurement unit. J. Appl. Biomech..

[42] Faber G.S., Kingma I., van Dieën J.H. (2010). Bottom-up estimation of joint moments during manual lifting using orientation sensors instead of position sensors. J. Biomech..

[43] Krüger A., McAlpine P., Borrani F., Edelmann-Nusser J. (2011). Determination of three-dimensional joint loading within the lower extremities in snowboarding. J. Eng. Med..

[44] Higgins J.P.T., Green S. (2008). Cochrane Handbook for Systematic Reviews of Interventions.

[45] Seshadri D.R., Drummond C., Craker J., Rowbottom J.R., Voos J.E. (2017). Wearable devices for sports: New integrated technologies allow coaches, physicians, and trainers to better understand the physical demands of athletes in real time. IEEE Pulse.

[46] Espinosa H.G., Lee J., James D.A. (2015). The inertial sensor: A base platform for wider adoption in sports science applications. J. Fit. Res..

[47] Armstrong S. (2007). Wireless connectivity for health and sports monitoring: A review. Br. J. Sports Med..

[48] Gabbett T.J. (2013). Quantifying the physical demands of collision sports. J. Strength Cond. Res..

[49] Mooney R., Corley G., Godfrey A., Quinlan L., ÓLaighin G. (2016). Inertial sensor technology for elite swimming performance analysis: A systematic review. Sensors.

[50] Picerno P. (2017). Good practice rules for the assessment of the force-velocity relationship in isoinertial resistance exercises. Asian J. Sports Med..

[51] Dadashi F., Millet G.P., Aminian K. (2013). Inertial measurement unit and biomechanial analysis of swimming: An update. Swiss J. Sports Med. Sports Traumatol..

[52] Ahmadi A., Mitchell E., Richter C., Destelle F., Gowing M., O’Connor N.E., Moran K. (2015). Towards automatic activity classification and movement assessment during a sports training session. IEEE Internet Things J..

[53] Ahmadi A., Rowlands D.D., James D.A. (2010). Development of inertial and novel marker-based techniques and analysis for upper arm rotational velocity measurements in tennis. Sports Eng..

[54] Ahmadi A., Rowlands D., James D.A. (2010). Towards a wearable device for skill assessment and skill acquisition of a tennis player during the first serve. Sports Eng..

[55] Akins J.S., Heebner N.R., Lovalekar M., Sell T.C. (2015). Reliability and validity of instrumented soccer equipment. J. Appl. Biomech..

[56] Alexander J.P., Hopkinson T.L., Wundersitz D.W.T., Serpell B.G., Mara J.K., Ball N.B. (2016). Validity of a wearable accelerometer device to measure average acceleration values during high-speed running. J. Strength Cond. Res..

[57] Ammann R., Taube W., Wyss T. (2016). Accuracy of PARTwear inertial sensor and Optojump optical measurement system for measuring ground contact time during running. J. Strength Cond. Res..

[58] Anderson R., Harrison A., Lyons G.M. (2005). Accelerometry-based feedback—Can it improve movement consistency and performance in rowing?. Sports Biomech..

[59] Andrew D.P.S., Chow J.W., Knudson D.V., Tillman M.D. (2003). Effect of ball size on player reaction and racket acceleration during the tennis volley. J. Sci. Med. Sport.

[60] Atkinson M., Rosalie S., Netto K. (2016). Physical demand of seven closed agility drills. Sports Biomech..

[61] Auvinet B., Gloria E., Renault G., Barrey E. (2002). Runner’s stride analysis: Comparison of kinematic and kinetic analyses under field conditions. Sci. Sports.

[62] Avvenuti M., Cesarini D., Cimino M.G.C.A. (2013). MARS, a multi-agent system for assessing rowers’ coordination via motion-based stigmergy. Sensors.

[63] Bächlin M., Tröster G. (2012). Swimming performance and technique evaluation with wearable acceleration sensors. Pervasive Mob. Comput..

[64] Balsalobre-Fernández C., Kuzdub M., Poveda-Ortiz P., Campo-Vecino J. (2016). Del Validity and reliability of the PUSH wearable device to measure movement velocity during the back squat exercise. J. Strength Cond. Res..

[65] Balsalobre-Fernández C., Tejero-Gonzalez C.M., Del Campo-Vecino J., Alonso-Curiel D. (2013). The effects of a maximal power training cycle on the strength, maximum power, vertical jump height and acceleration of high-level 400-meter hurdlers. J. Hum. Kinet..

[66] Barrett S., Midgley A.W., Towlson C., Garrett A., Portas M., Lovell R. (2016). Within-match PlayerLoad patterns during a simulated soccer match: Potential implications for unit positioning and fatigue management. Int. J. Sports Physiol. Perform..

[67] Barrett S., Midgley A., Lovell R. (2014). PlayerLoad™: Reliability, convergent validity, and influence of unit position during treadmill running. Int. J. Sports Physiol. Perform..

[68] Beanland E., Main L.C., Aisbett B., Gastin P., Netto K. (2014). Validation of GPS and accelerometer technology in swimming. J. Sci. Med. Sport.

[69] Bergamini E., Guillon P., Camomilla V., Pillet H., Skalli W., Cappozzo A. (2013). Trunk inclination estimate during the sprint start using an inertial measurement unit: A validation study. J. Appl. Biomech..

[70] Bergamini E., Picerno P., Pillet H., Natta F., Thoreux P., Camomilla V. (2012). Estimation of temporal parameters during sprint running using a trunk-mounted inertial measurement unit. J. Biomech..

[71] Bergamini E., Morelli F., Marchetti F., Vannozzi G., Polidori L., Paradisi F., Traballesi M., Cappozzo A., Delussu A.S. (2015). Wheelchair propulsion biomechanics in junior basketball players: A method for the evaluation of the efficacy of a specific training program. BioMed Res. Int..

[72] Bigelow E.M.R., Elvin N.G., Elvin A.A., Arnoczky S.P. (2013). Peak impact accelerations during track and treadmill running. J. Appl. Biomech..

[73] Boyd L.J., Ball K., Aughey R.J. (2013). Quantifying external load in australian football matches and training using accelerometers. Int. J. Sports Physiol. Perform..

[74] Boyd L.J., Ball K., Aughey R.J. (2011). The reliability of MinimaxX accelerometers for measuring physical activity in Australian football. Int. J. Sports Physiol. Perform..

[75] Brodie M., Walmsley A., Page W. (2008). Fusion motion capture: A prototype system using inertial measurement units and GPS for the biomechanical analysis of ski racing. Sports Technol..

[76] Brown N., Bichler S., Alt W. (2015). Detecting repetitions and time features in resistance training using triaxial accelerometry. Sports Technol..

[77] Buchheit M., Morin J. (2015). Assessing stride variables and vertical stiffness with GPS embedded accelerometers preliminary insights for the monitoring of neuromuscular fatigue on the field. J. Sports Sci. Med..

[78] Buddhadev H.H., Vingren J.L., Duplanty A.A., Hill D.W. (2012). Mechanisms underlying the reduced performance measures from using equipment with a counterbalance weight system. J. Strength Cond. Res..

[79] Buśko K., Staniak Z., Szark-Eckardt M., Nikolaidis P.T., Mazur-Rózycka J., Łach P., Michalski R., Gajewski J., Górski M. (2016). Measuring the force of punches and kicks among combat sport athletes using a modified punching bag with an embedded accelerometer. Acta Bioeng. Biomech..

[80] Callaway A.J. (2015). Measuring kinematic variables in front crawl swimming using accelerometers: A validation study. Sensors.

[81] Callaway A.J., Cobb J.E. (2012). Linear acceleration measurement utilizing inter-instrument synchronization: A comparison between accelerometers and motion-based tracking approaches. Meas. Phys. Educ. Exerc. Sci..

[82] Caruso J.F., Olson N.M., Taylor S.T., McLagan J.R., Shepherd C.M., Borgsmiller J.A., Mason M.L., Riner R.R., Gilliland L., Grisewold S. (2012). Front squat data reproducibility collected with a triple-axis accelerometer. J. Strength Cond. Res..

[83] Casartelli N., Müller R., Maffiuletti N.A. (2010). Validity and reliability of the Myotest accelerometric system for the assessment of vertical jump height. J. Strength Cond. Res..

[84] Castagna C., Ganzetti M., Ditroilo M., Giovannelli M., Rocchetti A., Mazi V. (2013). Concurrent validity of vertical jump performance assessment systems. J. Strength Cond. Res..

[85] Chakravorti N., Le Sage T., Slawson S.E., Conway P.P., West A.A. (2013). Design and implementation of an integrated performance monitoring tool for swimming to extract stroke information at real time. IEEE Trans. Hum.-Mach. Syst..

[86] Chandler P.T., Pinder S.J., Curran J.D., Gabbett T.J. (2014). Physical demands of training and competition in collegiate netball players. J. Strength Cond. Res..

[87] Chapman R.F., Laymon A.S., Wilhite D.P., McKenzie J.M., Tanner D.A., Stager J.M. (2012). Ground contact time as an indicator of metabolic cost in elite distance runners. Med. Sci. Sports Exerc..

[88] Chardonnens J., Favre J., Cuendet F., Gremion G., Aminian K. (2014). Measurement of the dynamics in ski jumping using a wearable inertial sensor-based system. J. Sports Sci..

[89] Chardonnens J., Favre J., Cuendet F., Gremion G., Aminian K. (2013). Characterization of lower-limbs inter-segment coordination during the take-off extension in ski jumping. Hum. Mov. Sci..

[90] Chardonnens J., Favre J., Cuendet F., Gremion G., Aminian K. (2013). A system to measure the kinematics during the entire ski jump sequence using inertial sensors. J. Biomech..

[91] Chardonnens J., Favre J., Le Callennec B., Cuendet F., Gremion G., Aminian K. (2012). Automatic measurement of key ski jumping phases and temporal events with a wearable system. J. Sports Sci..

[92] Charlton P.C., Kenneally-Dabrowski C., Sheppard J., Spratford W. (2017). A simple method for quantifying jump loads in volleyball athletes. J. Sci. Med. Sport.

[93] Choukou M.A., Laffaye G., Taiar R. (2014). Reliability and validity of an accele-rometric system for assessing vertical jumping performance. Biol. Sport.

[94] Chua J.J.C., Fuss F.K., Subic A. (2017). Activity identification and classification in wheelchair rugby using fractal dimensions. Sports Eng..

[95] Cockcroft J., Muller J., Scheffer C. (2016). Robust tracking of bicycle crank angles using magneto-inertial sensors, domain constraints and functional frame alignment techniques. J. Sports Eng. Technol..

[96] Cockcroft J., Muller J.H., Scheffer C. (2014). A novel complimentary filter for tracking hip angles during cycling using wireless inertial sensors and dynamic acceleration estimation. IEEE Sens. J..

[97] Comstock B.A., Solomon-Hill G., Flanagan S.D., Earp J.E., Luk H.-Y., Dobbins K.A., Dunn-Lewis C., Fragala M.S., Ho J.-Y., Hatfield D.L. (2011). Validity of the Myotest^®^ in measuring force and power production in the squat and bench press. J. Strength Cond. Res..

[98] Cormack S.J., Mooney M.G., Morgan W., McGuigan M.R. (2013). Influence of neuromuscular fatigue on accelerometer load in elite Australian football players. Int. J. Sports Physiol. Perform..

[99] Cormack S.J., Smith R.L., Mooney M.M., Young W.B., O’Brien B.J. (2014). Accelerometer load as a measure of activity profile in different standards of netball match play. Int. J. Sports Physiol. Perform..

[100] Crewther B.T., Cunningham D.J., Cook C., Owen N. (2011). Validating two systems for estimating force and power. Int. J. Sports Med..

[101] Cummins C., Orr R. (2015). Analysis of physical collisions in elite national rugby league match play. Int. J. Sports Physiol. Perform..

[102] Cunniffe B., Proctor W., Baker J.S., Davies B. (2009). An evaluation of the physiological demands of elite rugby union using global positioning system tracking software. J. Strength Cond. Res..

[103] Da Silva B.V.C., de Moura Simim M.A., Marocolo M., Franchini E., da Mota G.R. (2015). Optimal load for the peak power and maximal strength of the upper body in Brazilian Jiu-Jitsu athletes. J. Strength Cond. Res..

[104] Dadashi F., Millet G.P., Aminian K. (2015). A Bayesian approach for pervasive estimation of breaststroke velocity using a wearable IMU. Pervasive Mob. Comput..

[105] Dadashi F., Millet G.P., Aminian K. (2013). Gaussian process framework for pervasive estimation of swimming velocity with body-worn IMU. Electron. Lett..

[106] Dadashi F., Crettenand F., Millet G.P., Aminian K. (2012). Front-crawl instantaneous velocity estimation using a wearable inertial measurement unit. Sensors.

[107] Dadashi F., Crettenand F., Millet G.P., Seifert L., Komar J., Aminian K. (2013). Automatic front-crawl temporal phase detection using adaptive filtering of inertial signals. J. Sports Sci..

[108] Dadashi F., Millet G.P., Aminian K. (2016). Front-crawl stroke descriptors variability assessment for skill characterization. J. Sports Sci..

[109] Dalen T., Ingebrigtsen J., Ettema G., Hjelde G.H., Wisløff U. (2016). Player load, acceleration, and deceleration during forty-five competitive matches of elite soccer. J. Strength Cond. Res..

[110] Davey N., Anderson M., James D.A. (2008). Validation trial of an accelerometer-based sensor platform for swimming. Sports Technol..

[111] Davies M.J., Young W., Farrow D., Bahnert A. (2013). Comparison of small-sided games on agility demands in elite australian football. Int. J. Sports Physiol. Perform..

[112] Del Coso J., Portillo J., Salinero J.J., Lara B., Abian-Vicen J., Areces F. (2016). Caffeinated energy drinks improve high-speed running in elite field hockey players. Int. J. Sport Nutr. Exerc. Metab..

[113] Dieu O., Vanhelst J., Bui-Xuân G., Blondeau T., Fardy P.S., Mikulovic J. (2014). Relationship between tactics and energy expenditure according to level of experience in badminton. Percept. Mot. Skills.

[114] Dovgalecs V., Boulanger J., Seifert L., Orth D., Hérault R., Coeurjolly J.F., Davids K. (2014). Movement phase detection in climbing. Sports Technol..

[115] Dowling A.V., Favre J., Andriacchi T.P. (2011). A wearable system to assess risk for anterior cruciate ligament injury during jump landing: Measurements of temporal events, jump height, and sagittal plane kinematics. J. Biomech. Eng..

[116] Dubus G. (2012). Evaluation of four models for the sonification of elite rowing. J. Multimodal User Interfaces.

[117] Elvin N.G., Elvin A.A., Arnoczky S.P. (2007). Correlation between ground reaction force and tibial acceleration in vertical jumping. J. Appl. Biomech..

[118] Enders H., von Tscharner V., Nigg B.M. (2014). The effects of preferred and non-preferred running strike patterns on tissue vibration properties. J. Sci. Med. Sport.

[119] Fantozzi S., Giovanardi A., de Magalhaes F.A., Di Michele R., Cortesi M., Gatta G. (2016). Assessment of three-dimensional joint kinematics of the upper limb during simulated swimming using wearable inertial-magnetic measurement units. J. Sports Sci..

[120] Fasel B., Favre J., Chardonnens J., Gremion G., Aminian K. (2015). An inertial sensor-based system for spatio-temporal analysis in classic cross-country skiing diagonal technique. J. Biomech..

[121] Fasel B., Praz C., Kayser B., Aminian K. (2016). Measuring spatio-temporal parameters of uphill ski-mountaineering with ski-fixed inertial sensors. J. Biomech..

[122] Fasel B., Spörri J., Gilgien M., Boffi G., Chardonnens J., Müller E., Aminian K. (2016). Three-dimensional body and centre of mass kinematics in alpine ski racing using differential GNSS and inertial sensors. Remote Sens..

[123] Flores F.J., Sedano S., de Benito A.M., Redondo J.C. (2016). Validity and reliability of a 3-axis accelerometer for measuring weightlifting movements. Int. J. Sports Sci. Coach..

[124] Fulton S.K., Pyne D.B., Burkett B. (2009). Quantifying freestyle kick-count and kick-rate patterns in Paralympic swimming. J. Sports Sci..

[125] Fulton S.K., Pyne D.B., Burkett B. (2011). Optimizing kick rate and amplitude for Paralympic swimmers via net force measures. J. Sports Sci..

[126] Fulton S.K., Pyne D.B., Burkett B. (2009). Validity and reliability of kick count and rate in freestyle using inertial sensor technology. J. Sports Sci..

[127] Gabbett T., Gahan C. (2016). Repeated high-intensity effort activity in relation to tries scored and conceded during rugby league match-play. Int. J. Sports Physiol. Perform..

[128] Gabbett T.J. (2015). Relationship between accelerometer load, collisions, and repeated high-intensity effort activity in rugby league players. J. Strength Cond. Res..

[129] Gabbett T.J. (2013). Influence of the opposing team on the physical demands of elite rugby league match play. J. Strength Cond. Res..

[130] Gabbett T.J. (2012). Sprinting patterns of national rugby league competition. J. Strength Cond. Res..

[131] Gabbett T.J. (2014). Influence of playing standard on the physical demands of junior rugby league tournament match-play. J. Sci. Med. Sport.

[132] Gabbett T.J., Jenkins D.G., Abernethy B. (2012). Influence of wrestling on the physiological and skill demands of small-sided games. J. Strength Cond. Res..

[133] Gabbett T.J., Jenkins D.G., Abernethy B. (2011). Physical collisions and injury in professional rugby league match-play. J. Sci. Med. Sport.

[134] Gabbett T.J., Jenkins D.G., Abernethy B. (2012). Physical demands of professional rugby league training and competition using microtechnology. J. Sci. Med. Sport.

[135] Gabbett T.J., Abernethy B., Jenkins D.G. (2012). Influence of field size on the physiological and skill demands of small-sided games in junior and senior rugby league players. J. Strength Cond. Res..

[136] Gageler W.H., Wearing S., James D.A., Grove K. (2015). Automatic jump detection method for athlete monitoring and performance in volleyball. Int. J. Perform. Anal. Sport.

[137] Gallo T., Cormack S., Gabbett T., Williams M., Lorenzen C. (2015). Characteristics impacting on session rating of perceived exertion training load in Australian footballers. J. Sports Sci..

[138] Gandy E.A., Bondi A., Hogg R., Pigott T.M.C. (2014). A preliminary investigation of the use of inertial sensing technology for the measurement of hip rotation asymmetry in horse riders. Sports Technol..

[139] Gao Z., Song B., Liu M., Song G., Sun W., Ge Y. (2009). Design and application of a multidimensional acceleration sensor for coaching of shot-put athletes. Sens. Actuators A Phys..

[140] García-Pérez J.A., Pérez-Soriano P., Llana Belloch S., Lucas-Cuevas A.G., Sánchez-Zuriaga D., Lucas-Cuevas Á.G., Sánchez-Zuriaga D. (2014). Effects of treadmill running and fatigue on impact acceleration in distance running. Sports Biomech..

[141] Gašic T., Bubanj S., Živković M., Stanković R., Bubanj R., Obradović B. (2011). Difference in the explosive strength of upper extremities between athletes in relation to their sport activity, type of engagement in sport and gender. Sport Sci..

[142] Gastin P.B., Mclean O.C., Breed R.V.P., Spittle M. (2014). Tackle and impact detection in elite Australian football using wearable microsensor technology. J. Sports Sci..

[143] Gastin P.B., McLean O., Spittle M., Breed R.V.P. (2013). Quantification of tackling demands in professional Australian football using integrated wearable athlete tracking technology. J. Sci. Med. Sport.

[144] Gaudino P., Iaia F.M., Strudwick A.J., Hawkins R.D., Alberti G., Atkinson G., Gregson W. (2015). Factors influencing perception of effort (session-RPE) during elite soccer training. Int. J. Sports Physiol. Perform..

[145] Gescheit D.T., Cormack S.J., Reid M., Duffield R. (2015). Consecutive days of prolonged tennis match play: Performance, physical, and perceptual responses in trained players. Int. J. Sports Physiol. Perform..

[146] Ghasemzadeh H., Jafari R. (2011). Coordination analysis of human movements with body sensor networks: A signal processing model to evaluate baseball swings. IEEE Sens. J..

[147] Ghasemzadeh H., Loseu V., Jafari R. (2009). Wearable coach for sport training: A quantitative model to evaluate wrist-rotation in golf. J. Ambient Intell. Smart Environ..

[148] Giandolini M., Poupard T., Gimenez P., Horvais N., Millet G.Y., Morin J.B., Samozino P. (2014). A simple field method to identify foot strike pattern during running. J. Biomech..

[149] Gindre C., Lussiana T., Hebert-Losier K., Morin J.B. (2016). Reliability and validity of the Myotest^®^ for measuring running stride kinematics. J. Sports Sci..

[150] Giroux C., Rabita G., Chollet D., Guilhem G. (2014). What is the best method for assessing lower limb force-velocity relationship?. Int. J. Sports Med..

[151] Gomez-Piriz P.T., Sanchez E.T., Manrique D.C., Gonzalez E.P. (2012). Reliability and comparability of the accelerometer and the linear position measuring device in resistance training. J. Strength Cond. Res..

[152] Gonçalves B.V., Figueira B.E., Maçãs V., Sampaio J. (2014). Effect of player position on movement behaviour, physical and physiological performances during an 11-a-side football game. J. Sports Sci..

[153] Gouttebarge V., Wolfard R., Griek N., De Ruiter C.J., Boschman J.S., VanDieën J.H. (2015). Reproducibility and validity of the myotest for measuring step frequency and ground contact time in recreational runners. J. Hum. Kinet..

[154] Groh B.H., Flaschka J., Wirth M., Kautz T., Fleckenstein M., Eskofier B.M. (2016). Wearable real-time skateboard trick visualization and its community perception. IEEE Comput. Graph. Appl..

[155] Gullstrand L., Halvorsen K., Tinmark F., Eriksson M., Nilsson J. (2009). Measurements of vertical displacement in running, a methodological comparison. Gait Posture.

[156] Hagem R.M., O’Keefe S.G., Fickenscher T., Thiel D.V. (2013). Self contained adaptable optical wireless communications system for stroke rate during swimming. IEEE Sens. J..

[157] Hamill J., Derrick T.R., Holt K.G. (1995). Shock attenuation and stride frequency during running. Hum. Mov. Sci..

[158] Hang Y.S., Lippert F.G., Spolek G.A., Frankel V.H., Harrington R.M. (1979). Biomechanical study of the pitching elbow. Int. Orthop..

[159] Harding J.W., Mackintosh C.G., Martin D.T., Hahn A.G., James D.A. (2009). Automated scoring for elite half-pipe snowboard competition: Important sporting development or techno distraction?. Sports Technol..

[160] Hausswirth C., Le Meur Y., Couturier A., Bernard T., Brisswalter J. (2009). Accuracy and repeatability of the Polar^®^ RS800sd to evaluate stride rate and running speed. Int. J. Sports Med..

[161] Helten T., Brock H., Muller M., Seidel H.P. (2011). Classification of trampoline jumps using inertial sensors. Sports Eng..

[162] Herren R., Sparti A., Aminian K., Schutz Y. (1999). The prediction of speed and incline in outdoor running in humans using accelerometry. Med. Sci. Sports Exerc..

[163] Hoppe M., Baumgart C., Freiwald J. (2011). Do running activities of adolescent and adult tennis players differ during play?. Int. J. Sport Nutr. Exerc. Metab..

[164] Hou P. (2012). The study on swimming exercises based on 3D accelerometer data analysis. Int. J. Adv. Comput. Technol..

[165] Hurst H.T., Atkins S., Kirk C. (2014). Reliability of a portable accelerometer for measuring workload during mixed martial arts. J. Athl. Enhanc..

[166] Innocenti B., Facchielli D., Torti S., Verza A. (2006). Analysis of biomechanical quantitites during a squat jump: Evaluation of a performance index. J. Strength Cond. Res..

[167] Jaitner T., Schmidt M., Nolte K., Rheinlander C., Wille S., Wehn N. (2015). Vertical jump diagnosis for multiple athletes using a wearable inertial sensor unit. Sports Technol..

[168] James D.A., Leadbetter R.I., Neeli M.R., Burkett B.J., Thiel D.V., Lee J.B. (2011). An integrated swimming monitoring system for the biomechanical analysis of swimming strokes. Sports Technol..

[169] Janssen I., Sachlikidis A. (2010). Validity and reliability of intra-stroke kayak velocity and acceleration using a GPS-based accelerometer. Sports Biomech..

[170] Jarning J.M., Mok K.M., Hansen B.H., Bahr R. (2015). Application of a tri-axial accelerometer to estimate jump frequency in volleyball. Sports Biomech..

[171] Jensen U., Schmidt M., Hennig M., Dassler F.A., Jaitner T., Eskofier B.M. (2015). An IMU-based mobile system for golf putt analysis. Sports Eng..

[172] Jidovtseff B., Croisier J., Lhermerout C. (2006). The concept of iso-inertial assessment: Reproducibility analysis and descriptive data. Isokinet. Exerc. Sci..

[173] Jones I.C., Miller D.I. (1996). Influence of fulcrum position on springboard response and takeoff performance in the running approach. J. Appl. Biomech..

[174] Kawabata M., Goto K., Fukusaki C., Sasaki K., Hihara E., Mizushina T., Ishii N. (2013). Acceleration patterns in the lower and upper trunk during running. J. Sports Sci..

[175] Keaney E., Withers S., Parker-Simmons S., Gastin P., Netto K. (2016). The training load of aerial skiing. Int. J. Perform. Anal. Sport.

[176] Kelly D., Coughlan G.F., Green B.S., Caulfield B. (2012). Automatic detection of collisions in elite level rugby union using a wearable sensing device. Sports Eng..

[177] Kelly P., Conaire C.O., O’Connor N.E., Hodgins J. (2013). Motion synthesis for sports using unobtrusive lightweight body-worn and environment sensing. Comput. Graph. Forum.

[178] Kempton T., Sullivan C., Bilsborough J.C., Cordy J., Coutts A.J. (2015). Match-to-match variation in physical activity and technical skill measures in professional Australian Football. J. Sci. Med. Sport.

[179] King K., Hough J., McGinnis R., Perkins N.C. (2012). A new technology for resolving the dynamics of a swinging bat. Sports Eng..

[180] King K., Perkins N.C., Churchill H., McGinnis R., Doss R., Hickland R. (2011). Bowling ball dynamics revealed by miniature wireless MEMS inertial measurement unit. Sports Eng..

[181] King K., Yoon S.W., Perkins N.C., Najafi K. (2008). Wireless MEMS inertial sensor system for golf swing dynamics. Sens. Actuators A Phys..

[182] Kirk C., Hurst H.T., Atkins S. (2015). Measuring the workload of mixed martial arts using accelerometry, time motion analysis and lactate. Int. J. Perform. Anal. Sport.

[183] Kirk C., Hurst H., Atkins S. (2015). Comparison of the training loads of mixed martial arts techniques in isolated training and open sparring. J. Combat Sports Martial Arts.

[184] Kobsar D., Osis S.T., Hettinga B.A., Ferber R. (2014). Classification accuracy of a single tri-axial accelerometer for training background and experience level in runners. J. Biomech..

[185] Koda H., Sagawa K., Kuroshima K., Tsukamoto T., Urita K., Ishibashi Y. (2010). 3D measurement of forearm and upper arm during throwing motion using body mounted sensor. J. Adv. Mech. Des. Syst. Manuf..

[186] Koshida S., Urabe Y., Miyashita K., Iwai K., Kagimori A. (2008). Muscular outputs during dynamic bench press under stable versus unstable conditions. J. Strength Cond. Res..

[187] Križaj D., Čuk I. (2015). Can miniature accelerometers attached to the gymnastics springboard be used for take-off analysis?. Sci. Gymnast. J..

[188] Krüger A., Edelmann-Nusser J. (2009). Biomechanical analysis in freestyle snowboarding: Application of a full-body inertial measurement system and a bilateral insole measurement system. Sports Technol..

[189] Krüger A., Edelmann-Nusser J. (2010). Application of a full body inertial measurement system in alpine skiing: A comparison with an optical video based system. J. Appl. Biomech..

[190] Laffaye G., Collin J.M., Levernier G., Padulo J. (2014). Upper-limb power test in rock-climbing. Int. J. Sports Med..

[191] Le Bris R., Billat V., Auvinet B., Chaleil D., Hamard L., Barrey E. (2006). Effect of fatigue on stride pattern continuously measured by an accelerometric gait recorder in middle distance runners. J. Sports Med. Phys. Fit..

[192] Le Sage T., Conway P., Cossor J., Slawson S., West A. (2013). A wireless sensor system for monitoring the performance of a swimmer’s tumble turn. J. Sports Eng. Technol..

[193] Le Sage T., Conway P., Slawson S., West A. (2013). Development of a wireless sensor network for use as an automated system for monitoring swimming starts. J. Sports Eng. Technol..

[194] Lee J.B., Mellifont R.B., Burkett B.J. (2010). The use of a single inertial sensor to identify stride, step, and stance durations of running gait. J. Sci. Med. Sport.

[195] Lee J.B., Mellifont R.B., Burkett B.J., James D.A. (2013). Detection of illegal race walking: A tool to assist coaching and judging. Sensors.

[196] Lee J.B., Sutter K.J., Askew C.D., Burkett B.J. (2010). Identifying symmetry in running gait using a single inertial sensor. J. Sci. Med. Sport.

[197] Lee J., Leadbetter R., Ohgi Y., Thiel D., Burkett B., James D.A. (2011). Quantifying and assessing biomechanical differences in swim turn using wearable sensors. Sports Technol..

[198] Leite M.A., Sasaki J.E., Lourenço C.L.M., Zanetti H.R., Cruz L.G., da Mota G.R., Mendes E.L. (2016). Medicine ball throw test predicts arm power in rugby sevens players. Rev. Bras. Cineantropometria Desempenho Hum..

[199] Lesinski M., Muehlbauer T., Granacher U. (2016). Concurrent validity of the Gyko inertial sensor system for the assessment of vertical jump height in female sub-elite youth soccer players. BMC Sports Sci. Med. Rehabil..

[200] Lin S.P., Sung W.H., Kuo T.B.J., Chen J.J. (2015). Development and application of gravity acceleration measurement in running kinematic analysis. ICIC Express Lett..

[201] Lin S.-P., Sung W.-H., Kuo F.-C., Kuo T.B.J., Chen J.-J. (2014). Impact of center-of-mass acceleration on the performance of ultramarathon runners. J. Hum. Kinet..

[202] Lindsay T.R., Noakes T.D., McGregor S.J. (2014). Effect of treadmill versus overground running on the structure of variability of stride timing. Percept. Mot. Skills.

[203] Lindsay T.R., Yaggie J.A., McGregor S.J. (2016). A wireless accelerometer node for reliable and valid measurement of lumbar accelerations during treadmill running. Sports Biomech..

[204] Little C., Lee J.B.J.J.B., James D.A., Davison K. (2013). An evaluation of inertial sensor technology in the discrimination of human gait. J. Sports Sci..

[205] Llosa J., Vilajosana I., Vilajosana X., Navarro N., Suriach E., Marqus J.M. (2009). REMOTE, a wireless sensor network based system to monitor rowing performance. Sensors.

[206] Lomond K.V., Turcotte R.A., Pearsall D.J. (2007). Three-dimensional analysis of blade contact in an ice hockey slap shot, in relation to player skill. Sports Eng..

[207] Losnegard T., Myklebust H., Skattebo O., Stadheim H.K., Sandbakk Ø., Hallén J. (2017). The influence of pole length on performance, O_2_ cost, and kinematics in double poling. Int. J. Sports Physiol. Perform..

[208] Lovell T.W.J., Sirotic A.C., Impellizzeri F.M., Coutts A.J. (2013). Factors affecting perception of effort (session rating of perceived exertion) during rugby league training. Int. J. Sports Physiol. Perform..

[209] Lowery R.P., Joy J.M., Brown L.E., Oliveira de Souza E., Wistocki D.R., Davis G.S., Naimo M.A., Zito G.A., Wilson J.M. (2014). Effects of static stretching on 1-mile uphill run performance. J. Strength Cond. Res..

[210] Luteberget L.S., Spencer M. (2017). High-intensity events in international women’s team handball matches. Int. J. Sports Physiol. Perform..

[211] Macdermid P.W., Fink P.W., Stannard S.R. (2014). Transference of 3D accelerations during cross country mountain biking. J. Biomech..

[212] Macdermid P.W., Fink P.W., Stannard S.R. (2015). The effects of vibrations experienced during road vs off-road cycling. Int. J. Sports Med..

[213] Magnúsdóttir Á., Þorgilsson B., Karlsson B. (2014). Comparing three devices for jump height measurement in a heterogeneous group of subjects. J. Strength Cond. Res..

[214] Marin-Perianu R., Marin-Perianu M., Havinga P., Taylor S., Begg R., Palaniswami M., Rouffet D. (2013). A performance analysis of a wireless body-area network monitoring system for professional cycling. Pers. Ubiquitous Comput..

[215] Marsland F., Lyons K., Anson J., Waddington G., Macintosh C., Chapman D. (2012). Identification of cross-country skiing movement patterns using micro-sensors. Sensors.

[216] Mason B.S., Rhodes J.M., Goosey-Tolfrey V.L. (2014). Validity and reliability of an inertial sensor for wheelchair court sports performance. J. Appl. Biomech..

[217] Mattes K., Schaffert N. (2010). New measuring and on water coaching device for rowing. J. Hum. Sport Exerc..

[218] Mauch M., Rennbahn P.A., nat Hans-Joachim Rist R., Xaver Kaelin M. (2014). Reliability and validity of two measurement systems in the quantification of jump performance. Schweiz. Z. Sportmed. Sporttraumatol..

[219] McCurdy K.W., Walker J.L., Langford G.A., Kutz M.R., Guerrero J.M., McMillan J. (2010). The relationship between kinematic determinants of jump and sprint performance in division I women soccer players. J. Strength Cond. Res..

[220] McGinnis R.S., Cain S.M., Davidson S.P., Vitali R.V., Perkins N.C., McLean S.G. (2016). Quantifying the effects of load carriage and fatigue under load on sacral kinematics during countermovement vertical jump with IMU-based method. Sports Eng..

[221] McGinnis R.S., Perkins N.C. (2012). A highly miniaturized, wireless inertial measurement unit for characterizing the dynamics of pitched baseballs and softballs. Sensors.

[222] McGrath D., Greene B.R., O’Donovan K.J., Caulfield B. (2012). Gyroscope-based assessment of temporal gait parameters during treadmill walking and running. Sports Eng..

[223] McGregor S.J., Busa M.A., Skufca J., Yaggie J.A., Bollt E.M. (2009). Control entropy identifies differential changes in complexity of walking and running gait patterns with increasing speed in highly trained runners. Chaos.

[224] McGregor S.J., Busa M.A., Yagie J.A., Bollt E.M. (2009). High resolution MEMS accelerometers to estimate VO2 and compare running mechanics between highly trained inter-collegiate and untrained runners. PLoS ONE.

[225] McLellan C.P., Lovell D.I. (2012). Neuromuscular responses to impact and collision during elite rugby league match play. J. Strength Cond. Res..

[226] McLellan C.P., Lovell D.I., Gass G.C. (2011). Performance analysis of elite rugby league match play using global positioning systems. J. Strength Cond. Res..

[227] McNamara D.J., Gabbett T.J., Chapman P., Naughton G., Farhart P. (2015). The validity of microsensors to automatically detect bowling events and counts in cricket fast bowlers. Int. J. Sports Physiol. Perform..

[228] Meamarbashi A., Hossaini S.R.A. (2010). Application of novel inertial technique to compare the kinematics and kinetics of the legs in the soccer instep kick. J. Hum. Kinet..

[229] Mercer J.A., Devita P., Derrick T.R., Bates B.T. (2003). Individual effects of stride length and frequency on shock attenuation during running. Med. Sci. Sports Exerc..

[230] Mercer J.A., Vance J., Hreljac A., Hamill J. (2002). Relationship between shock attenuation and stride length during running at different velocities. Eur. J. Appl. Physiol..

[231] Michahelles F., Schiele B. (2005). Sensing and monitoring professional skiers. IEEE Pervasive Comput..

[232] Mitchell E., Monaghan D., O’Connor N.E. (2013). Classification of sporting activities using smartphone accelerometers. Sensors.

[233] Monnet T., Decatoire A., Lacouture P. (2014). Comparison of algorithms to determine jump height and flight time from body mounted accelerometers. Sports Eng..

[234] Montgomery P.G., Pyne D.B., Minahan C.L. (2010). The physical and physiological demands of basketball training and competition. Int. J. Sports Physiol. Perform..

[235] Moreira G.P., de Oliveira C.I.J., Greco P.J. (2015). Numerical superiority changes the physical demands of soccer players during small-sided games. Rev. Bras. Cineantropometria Desempenho Hum..

[236] Myers D. (2012). Relationship of anthropometric measurements and body composition to upper-body power in baseball players. Mo. J. Health Phys. Educ. Recreat. Dance.

[237] Myklebust H., Losnegard T., Hallén J. (2014). Differences in V1 and V2 ski skating techniques described by accelerometers. Scand. J. Med. Sci. Sports.

[238] Najafi B., Lee-Eng J., Wrobel J.S., Goebel R. (2015). Estimation of center of mass trajectory using wearable sensors during golf swing. J. Sports Sci. Med..

[239] Nakano G., Iino Y., Imura A., Kojima T. (2014). Transfer of momentum from different arm segments to a light movable target during a straight punch thrown by expert boxers. J. Sports Sci..

[240] Nedergaard N.J., Robinson M.A., Eusterwiemann E., Drust B., Lisboa P.J., Vanrenterghem J. (2017). The relationship between whole-body external loading and body-worn accelerometry during team-sport movements. Int. J. Sports Physiol. Perform..

[241] Nedergaard N.J., Kersting U., Lake M. (2014). Using accelerometry to quantify deceleration during a high-intensity soccer turning manoeuvre. J. Sports Sci..

[242] Neville J.G., Rowlands D.D., Lee J.B., James D.A. (2016). A model for comparing over-ground running speed and accelerometer derived step rate in elite level athletes. IEEE Sens. J..

[243] Norris M., Kenny I.C., Anderson R. (2016). Comparison of accelerometry stride time calculation methods. J. Biomech..

[244] Nuzzo J.L., Anning J.H., Scharfenberg J.M. (2011). The reliability of three devices used for measuring vertical jump height. J. Strength Cond. Res..

[245] Connor S.O., McCaffrey N., Whyte E., Moran K. (2016). The novel use of a SenseCam and accelerometer to validate training load and training information in a self-recall training diary. J. Sports Sci..

[246] Ohgi Y., Ichikawa H., Homma M., Miyaji C. (2003). Stroke phase discrimination in breaststroke swimming using a tri-axial acceleration sensor device. Sports Eng..

[247] Ohgi Y., Ichikawa H., Miyaji C. (2002). Microcomputer-based acceleration sensor device for swimming stroke monitoring. JSME Int. J. Ser. C Mech. Syst. Mach. Elem. Manuf..

[248] Omkar S.N., Vanjare A.M., Suhith H., Kumar G.S. (2012). Motion analysis for short and long jump. Int. J. Perform. Anal. Sport.

[249] Page R., Marrin K., Brogden C., Greig M. (2015). Biomechanical and physiological response to a contemporary soccer match-play simulation. J. Strength Cond. Res..

[250] Pansiot J., Zhang Z., Lo B., Yang G. (2011). WISDOM: Wheelchair inertial sensors for displacement and orientation monitoring. Meas. Sci. Technol..

[251] Picerno P., Camomilla V., Capranica L. (2011). Countermovement jump performance assessment using a wearable 3D inertial measurement unit. J. Sports Sci..

[252] Picerno P., Iannetta D., Comotto S., Donati M., Pecoraro F., Zok M., Tollis G., Figura M., Varalda C., Di Muzio D. (2016). 1RM prediction: A novel methodology based on the force-velocity and load-velocity relationships. Eur. J. Appl. Physiol..

[253] Polgaze T., Dawson B., Hiscock D.J., Peeling P. (2015). A comparative analysis of acceleromter and time motion data in elite mens hockey training and competition. Int. J. Sports Physiol. Perform..

[254] Praz C., Fasel B., Vuistiner P., Aminian K., Kayser B. (2016). Optimal slopes and speeds in uphill ski mountaineering: A laboratory study. Eur. J. Appl. Physiol..

[255] Praz C., Fasel B., Vuistiner P., Aminian K., Kayser B. (2016). Optimal slopes and speeds in uphill ski mountaineering: A field study. Eur. J. Appl. Physiol..

[256] Pruyn E.C., Watsford M.L., Murphy A.J. (2015). Differences in lower-body stiffness between levels of netball competition. J. Strength Cond. Res..

[257] Requena B., Garcia I., Requena F., Bressel E., Saez-Saez de Villarreal E., Cronin J. (2014). Association between traditional standing vertical jumps and a soccer-specific vertical jump. Eur. J. Sport Sci..

[258] Requena B., Requena F., García I., de Villarreal E.S.S., Pääsuke M. (2012). Reliability and validity of a wireless microelectromechanicals based system (Keimove^TM^) for measuring vertical jumping performance. J. Sports Sci. Med..

[259] Ritchie D., Hopkins W.G., Buchheit M., Cordy J., Bartlett J.D. (2016). Quantification of training and competition load across a season in an elite Australian Football club. Int. J. Sports Physiol. Perform..

[260] Robinson M.G., Holt L.E., Pelham T.W., Furneaux K. (2011). Accelerometry measurements of sprint kayaks: The coaches’ new tool. Int. J. Coach. Sci..

[261] Rontu J.P., Hannula M.I., Leskinen S., Linnamo V., Salmi J.A. (2010). One-repetition maximum bench press performance estimated with a new accelerometer method. J. Strength Cond. Res..

[262] Rowlands A.V., Stone M.R., Eston R.G. (2007). Influence of speed and step frequency during walking and running on motion sensor output. Med. Sci. Sports Exerc..

[263] Rowlands D.D., James D.A., Lee J.B. (2013). Visualization of wearable sensor data during swimming for performance analysis. Sports Technol..

[264] Rowlands D.D., James D.A., Thiel D. (2009). Bowler analysis in cricket using centre of mass inertial monitoring. Sports Technol..

[265] Ruben R.M., Molinari M.A., Bibbee C.A., Childress M.A., Harman M.S., Reed K.P., Haff G.G. (2010). The acute effects of an ascending squat protocol on performance during horizontal plyometric jumps. J. Strength Cond. Res..

[266] Sadi F., Klukas R. (2013). New jump trajectory determination method using low-cost MEMS sensor fusion and augmented observations for GPS/INS integration. GPS Solut..

[267] Sagawa K., Abo S., Tsukamoto T., Kondo I. (2009). Forearm trajectory measurement during pitching motion using an elbow-mounted sensor. J. Adv. Mech. Des. Syst. Manuf..

[268] Sakurai Y., Zenya F., Ishige Y. (2014). Automated identification and evaluation of subtechniques in classical-style roller skiing. J. Sports Sci. Med..

[269] Saponara S. (2017). Wearable biometric performance measurement system for combat sports. IEEE Trans. Instrum. Meas..

[270] Sarkka O., Nieminen T., Suuriniemi S., Kettunen L. (2016). Augmented inertial measurements for analysis of javelin throwing mechanics. Sports Eng..

[271] Sato K., Sands W.A., Stone M.H. (2012). The reliability of accelerometry to measure weightlifting performance. Sports Biomech..

[272] Sato K., Smith S.L., Sands W.A. (2009). Validation of an accelerometer for measuring sport performance. J. Strength Cond. Res..

[273] Sbriccoli P., Camomilla V., Di Mario A., Quinzi F., Figura F., Felici F. (2010). Neuromuscular control adaptations in elite athletes: The case of top level karateka. Eur. J. Appl. Physiol..

[274] Scanlan A.T., Wen N., Tucker P.S., Dalbo V.J. (2014). The relationships between Internal and external training load models during basketball. J. Strength Cond. Res..

[275] Schaffert N., Mattes K. (2011). Designing an acoustic feedback system for on-water rowing training. Int. J. Comput. Sci. Sport.

[276] Schütte K.H., Maas E.A., Exadaktylos V., Berckmans D., Venter R.E., Vanwanseele B. (2015). Wireless tri-axial trunk accelerometry detects deviations in dynamic center of mass motion due to running-induced fatigue. PLoS ONE.

[277] Scott B.R., Lockie R.G., Knight T.J., Clark A.C., De Jonge X.A.K.J. (2013). A comparison of methods to quantify the in-season training load of professional soccer players. Int. J. Sports Physiol. Perform..

[278] Seifert L., Dovgalecs V., Boulanger J., Orth D., Hérault R., Davids K. (2014). Full-body movement pattern recognition in climbing. Sports Technol..

[279] Seifert L., Orth D., Boulanger J., Dovgalecs V., Hérault R., Davids K. (2014). Climbing skill and complexity of climbing wall design: Assessment of jerk as a novel indicator of performance fluency. J. Appl. Biomech..

[280] Seifert L., Wattebled L., Orth D., L’Hermette M., Boulanger J., Davids K. (2016). Skill transfer specificity shapes perception and action under varying environmental constraints. Hum. Mov. Sci..

[281] Setuain I., Martinikorena J., Gonzalez-Izal M., Martinez-Ramirez A., Gómez M., Alfaro-Adrián J., Izquierdo M. (2016). Vertical jumping biomechanical evaluation through the use of an inertial sensor-based technology. J. Sports Sci..

[282] Shepherd J.B., Wada T., Rowlands D., James D.A. (2016). A novel AHRS inertial sensor-based algorithm for wheelchair propulsion performance analysis. Algorithms.

[283] Sinclair J., Toth J., Hobbs S.J. (2015). The influence of energy return and minimalist footwear on the kinetics and kinematics of depth jumping in relation to conventional trainers. Kinesiology.

[284] Siska L., Brodáni J., Stefanovský M., Todorov S. (2016). Basic reliability parameters of a boxing punch. J. Phys. Educ. Sport.

[285] Slawson S.E., Justham L.M., Conway P.P., Le-Sage T., West A.A. (2012). Characterizing the swimming tumble turn using acceleration data. J. Sports Eng. Technol..

[286] Sleivert G., Taingahue M. (2004). The relationship between maximal jump-squat power and sprint acceleration in athletes. Eur. J. Appl. Physiol..

[287] Smith R.M., Loschner C. (2002). Biomechanics feedback for rowing. J. Sports Sci..

[288] Spratford W., Portus M., Wixted A., Leadbetter R., James D.A. (2015). Peak outward acceleration and ball release in cricket. J. Sports Sci..

[289] Squadrone R., Rodano R., Preatoni E. (2012). Comparison of velocity and power output data derived from an inertial based system and an optical encoder during squat lifts in a weight room setting. J. Sports Med. Phys. Fit..

[290] Stamm A., James D.A., Thiel D.V. (2013). Velocity profiling using inertial sensors for freestyle swimming. Sports Eng..

[291] Staniak Z., Buśko K., Górski M., Pastuszak A. (2016). Accelerometer profile of motion of the pelvic girdle in breaststroke swimming. J. Hum. Kinet..

[292] Starrs P., Chohan A., Fewtrell D., Richards J., Selfe J. (2012). Biomechanical differences between experienced and inexperienced wheelchair users during sport. Prosthet. Orthot. Int..

[293] Stetter B.J., Buckeridge E., Von Tscharner V., Nigg S.R., Nigg B.M. (2016). A novel approach to determine strides, ice contact, and swing phases during ice hockey skating using a single accelerometer. J. Appl. Biomech..

[294] Stickford A.S.L., Chapman R.F., Johnston J.D., Stager J.M. (2015). Lower-leg compression, running mechanics, and economy in trained distance runners. Int. J. Sports Physiol. Perform..

[295] Strohrmann C., Harms H., Kappeler-setz C., Tr G. (2012). Monitoring kinematic changes with fatigue in running using body-worn sensors. IEEE Trans. Inf. Technol. Biomed..

[296] Suarez-Arrones L., Arenas C., López G., Requena B., Terrill O., Mendez-Villanueva A. (2014). Positional differences in match running performance and physical collisions in men rugby sevens. Int. J. Sports Physiol. Perform..

[297] Suarez-Arrones L., Portillo J., Pareja-Blanco F., De Villareal E.S., Sánchez-Medina L., Munguía-Izquierdo D. (2014). Match-play activity profile in elite women’s rugby union players. J. Strength Cond. Res..

[298] Sullivan C., Bilsborough J.C., Cianciosi M., Hocking J., Cordy J., Coutts A.J. (2014). Match score affects activity profile and skill performance in professional Australian Football players. J. Sci. Med. Sport.

[299] Sullivan C., Bilsborough J.C., Cianciosi M., Hocking J., Cordy J.T., Coutts A.J. (2014). Factors affecting match performance in professional australian football. Int. J. Sports Physiol. Perform..

[300] Supej M. (2010). 3D measurements of alpine skiing with an inertial sensor motion capture suit and GNSS RTK system. J. Sports Sci..

[301] Takano K., Li K.F. (2013). A multimedia tennis instruction system: Tracking and classifying swing motions. Int. J. Space Based Situated Comput..

[302] Tan H., Wilson A.M., Lowe J. (2008). Measurement of stride parameters using a wearable GPS and inertial measurement unit. J. Biomech..

[303] Tang W.-T., Zhang W.-Y., Huang C.-C., Young M.-S., Hwang I.-S. (2008). Postural tremor and control of the upper limb in air pistol shooters. J. Sports Sci..

[304] Thomas O., Sunehag P., Dror G., Yun S., Kim S., Robards M., Smola A., Green D., Saunders P. (2010). Wearable sensor activity analysis using semi-Markov models with a grammar. Pervasive Mob. Comput..

[305] Thompson C.J., Bemben M.G. (1999). Reliability and comparability of the accelerometer as a measure of muscular power. Med. Sci. Sports Exerc..

[306] Vales-Alonso J., Chaves-Dieguez D., Lopez-Matencio P., Alcaraz J.J., Parrado-Garcia F.J., Gonzalez-Castano F.J. (2015). SAETA: A smart coaching assistant for professional volleyball training. IEEE Trans. Syst. Man Cybern. Syst..

[307] Van der Slikke R.M.A., Berger M.A.M., Bregman D.J.J., Lagerberg A.H., Veeger H.E.J. (2015). Opportunities for measuring wheelchair kinematics in match settings; reliability of a three inertial sensor configuration. J. Biomech..

[308] Van der Slikke R.M.A., Berger M.A.M., Bregman D.J.J., Veeger H.E.J. (2016). From big data to rich data: The key features of athlete wheelchair mobility performance. J. Biomech..

[309] Villaseñor A., Turcotte R.A., Pearsall D.J. (2006). Recoil effect of the ice hockey stick during a slap shot. J. Appl. Biomech..

[310] Vingren J.L., Buddhadev H.H., Hill D.W. (2011). Smith machine counterbalance system affects measures of maximal bench press throw performance. J. Strength Cond. Res..

[311] Waldron M., Worsfold P.R., Twist C., Lamb K. (2014). A three-season comparison of match performances among selected and unselected elite youth rugby league players. J. Sports Sci..

[312] Waldron M., Worsfold P., Twist C., Lamb K. (2011). Concurrent validity and test-retest reliability of a global positioning system (GPS) and timing gates to assess sprint performance variables. J. Sports Sci..

[313] Warlop T.B., Bollens B., Crevecoeur F., Detrembleur C., Lejeune T.M. (2013). Dynamics of revolution time variability in cycling pattern: Voluntary intent can alter the long-range autocorrelations. Ann. Biomed. Eng..

[314] Watanabe K., Hokari M. (2006). Kinematical analysis and measurement of sports form. IEEE Trans. Syst. Man Cybern. Part A Syst. Hum..

[315] Watari R., Hettinga B., Osis S., Ferber R. (2016). Validation of a torso-mounted accelerometer for measures of vertical oscillation and ground contact time during treadmill running. J. Appl. Biomech..

[316] Weaving D., Marshall P., Earle K., Nevill A.M., Abt G. (2014). Combining internal- and external-training-load measures in professional rugby league. Int. J. Sports Physiol. Perform..

[317] Wellman A.D., Coad S.C., Goulet G.C., McLellan C.P. (2016). Quantification of competitive game demands of NCAA division I college football players using global positioning systems. J. Strength Cond. Res..

[318] Weyand P.G., Kelly M., Blackadar T., Darley J.C., Oliver S.R., Ohlenbusch N.E., Joffe S.W., Hoyt R.W. (2001). Ambulatory estimates of maximal aerobic power from foot-ground contact times and heart rates in running humans. J. Appl. Physiol..

[319] White A., MacFarlane N. (2015). Analysis of international competition and training in men’s field hockey by global positioning system and inertial sensor technology. J. Strength Cond. Res..

[320] Winter S.C., Lee J.B., Leadbetter R.I., Gordon S.J. (2016). Validation of a single inertial sensor for measuring running kinematics overground during a prolonged run. J. Fit. Res..

[321] Wixted A.J., Billing D.C., James D.A. (2010). Validation of trunk mounted inertial sensors for analysing running biomechanics under field conditions, using synchronously collected foot contact data. Sports Eng..

[322] Wixted A., Portus M., Spratford W., James D. (2011). Detection of throwing in cricket using wearable sensors. Sports Technol..

[323] Wright B.V., Stager J.M. (2013). Quantifying competitive swim training using accelerometer-based activity monitors. Sports Eng..

[324] Wundersitz D.W.T., Gastin P.B., Robertson S., Davey P.C., Netto K.J. (2015). Validation of a trunk-mounted accelerometer to measure peak impacts during team sport movements. Int. J. Sports Med..

[325] Wundersitz D.W.T., Gastin P.B., Richter C., Robertson S.J., Netto K.J. (2015). Validity of a trunk-mounted accelerometer to assess peak accelerations during walking, jogging and running. Eur. J. Sport Sci..

[326] Wundersitz D.W.T., Josman C., Gupta R., Netto K.J., Gastin P.B., Robertson S. (2015). Classification of team sport activities using a single wearable tracking device. J. Biomech..

[327] Wunsch T., Kröll J., Stöggl T., Schwameder H. (2017). Effects of a structured midsole on spatio-temporal variables and running economy in overground running. Eur. J. Sport Sci..

[328] Yang S., Mohr C., Li Q. (2011). Ambulatory running speed estimation using an inertial sensor. Gait Posture.

[329] Yiou E., Do M.C. (2001). In a complex sequential movement, what component of the motor program is improved with intensive practice, sequence timing or ensemble motor learning?. Exp. Brain Res..

[330] Zhang Y., Chen K., Yi J. (2013). Rider trunk and bicycle pose estimation with fusion of force/inertial sensors. IEEE Trans. Biomed. Eng..

[331] Zhang Y., Chen K., Yi J., Liu T., Pan Q. (2016). Whole-body pose estimation in human bicycle riding using a small set of wearable sensors. IEEE/ASME Trans. Mechatron..

[332] Zhao Y., Gerhard D., Barden J. (2015). Periodicity-based swimming performance feature extraction and parameter estimation. Sports Eng..

[333] Zihajehzadeh S., Loh D., Lee T.J., Hoskinson R., Park E.J. (2015). A cascaded Kalman filter-based GPS/MEMS-IMU integration for sports applications. Measurement.

[334] Zorko M., Nemec B., Babič J., Lešnik B., Supej M. (2015). The waist width of skis influences the kinematics of the knee joint in alpine skiing. J. Sports Sci. Med..

[335] Yuan Q., Chen I.M. (2012). Human velocity and dynamic behavior tracking method for inertial capture system. Sens. Actuators A Phys..

[336] Kelly S.J., Murphy A.J., Watsford M.L., Austin D., Rennie M. (2015). Reliability and validity of sports accelerometers during static and dynamic testing. Int. J. Sports Physiol. Perform..

[337] Abernethy P., Wilson G., Logan P. (1995). Strength and power assessment. Issues, controversies and challenges. Sports Med..

[338] Baker D., Wilson G., Carlyon B. (1994). Generality versus specificity: A comparison of dynamic and isometric measures of strength and speed-strength. Eur. J. Appl. Physiol. Occup. Physiol..

[339] Bosco C., Belli A., Astrua M., Tihanyi J., Pozzo R., Kellis S., Tsarpela O., Foti C., Manno R., Tranquilli C. (1995). A dynamometer for evaluation of dynamic muscle work. Eur. J. Appl. Physiol. Occup. Physiol..

[340] Roe M., Malone S., Blake C., Collins K., Gissane C., Büttner F., Murphy J.C., Delahunt E. (2017). A six stage operational framework for individualising injury risk management in sport. Inj. Epidemiol..

[341] Halson S.L. (2014). Monitoring training load to understand fatigue in athletes. Sports Med..

[342] #SportTechieTrends: 80+ Industry Experts Share the Sports Technology Trends and Innovations to Watch in 2017. https://www.sporttechie.com/sporttechietrends-80-industry-experts-share-the-sports-technology-trends-and-innovations-to-watch-in-2017/.

[343] Statistics & Facts on Wearable Technology. https://www.statista.com/topics/1556/wearable-technology/.

[344] Zervos H., Chansin G., Hayward J. (2017). Wearable Technology 2017–2027: Markets, Players, Forecasts: IDTechEx.

[345] Bughin L., Chui M., Manyika J. (2013). Ten IT-Enabled Business Trends for the Decade Ahead.

[346] Fuss F.K. (2009). Instrumentation of athletes and equipment during competitions. Sports Technol..

[347] Lightman K. (2016). Silicon gets sporty. IEEE Spectr..

[348] Hynes G., O’Grady M., O’Hare G. (2013). Towards accessible technologies for coaching. Int. J. Sports Sci. Coach..

[349] Mooney R., Corley G., Godfrey A., Osborough C., Newell J., Quinlan L.R., ÓLaighin G. (2016). Analysis of swimming performance: Perceptions and practices of US-based swimming coaches. J. Sports Sci..

[350] Ringuet-Riot C.J., Hahn A., James D.A. (2013). A structured approach for technology innovation in sport. Sports Technol..

[351] Baca A., Dabnichki P., Heller M., Kornfeind P. (2009). Ubiquitous computing in sports: A review and analysis. J. Sports Sci..

[352] Ringuet-Riot C., Carter S., James D.A. (2014). Programmed innovation in team sport using needs driven innovation. Procedia Eng..

[353] Zok M. Inertial sensors are changing the games. Proceedings of the 2014 International Symposium on Inertial Sensors and Systems (ISISS).

[354] Wells D., Cereatti A., Camomilla V., Donnelly C., Elliott B., Alderson J. A calibration procedure for MIMU sensors allowing for the calculation of elbow angles. Proceedings of the 33rd Annual Conference of the International Society of Biomechanics in Sport.

[355] Hummel O., Fehr U., Ferger K. (2013). Beyond ibeer-exploring the potential of smartphone sensors for performance diagnostics in sports. Int. J. Comput. Sci. Sport.

[356] Oliveira D.S., Afonso J.A., Ao S., Chan A.H., Katagiri H., Xu L. (2016). A smartphone-based multi-sensor wireless platform for cycling performance monitoring. IAENG Transactions on Engineering Sciences: Special Issue for the International Association of Engineers Conferences 2015.

[357] Baca A., Kornfeind P., Preuschl E., Bichler S., Tampier M., Novatchkov H. (2010). A server-based mobile coaching system. Sensors.

[358] Andreoni G., Standoli C.E., Paolo P.P. (2016). Defining requirements and related methods for designing sensorized garments. Sensors.

[359] Vasilyev P., Pearson S., El-Gohary M., Aboy M., McNames J. (2017). Inertial and time-of-arrival ranging sensor fusion. Gait Posture.

[360] Sabatini A., Genovese V. (2014). A sensor fusion method for tracking vertical velocity and height based on inertial and barometric altimeter measurements. Sensors.

[361] Son Y., Oh S. A barometer-IMU fusion method for vertical velocity and height estimation. Proceedings of the 2015 IEEE SENSORS.

[362] Liebermann D.G., Katz L., Hughes M.D., Bartlett R.M., McClements J., Franks I.M. (2002). Advances in the application of information technology to sport performance. J. Sports Sci..

[363] Umek A., Zhang Y., Tomažič S., Kos A. (2017). Suitability of strain gage sensors for integration into smart sport equipment: A golf club example. Sensors.

[364] Vales-Alonso J., Ĺopez-Matencio P., Gonzalez-Castãno F.J., Navarro-Helĺin H., Bãnos-Guirao P.J., Perez-Martinez F.J., Martinez-Álvarez R.P., González-Jiménez D., Gil-Castiñeira F., Duro-Ferńandez R. (2010). Ambient intelligence systems for personalized sport training. Sensors.

[365] James D.A., Fanella L., Cusani R. (2013). Near Real Time Network Simulation for Team Sports Monitoring. Procedia Eng..

[366] Ghasemzadeh H., Barnes J., Guenterberg E., Jafari R. A phonological expression for physical movement monitoring in body sensor networks. Proceedings of the 2008 5th IEEE International Conference on Mobile Ad-Hoc and Sensor Systems (MASS 2008).

[367] Baca A. (2014). Adaptive systems in sports. Social Networks and the Economics of Sports.

[368] Cormie P., McBride J.M., McCaulley G.O. (2007). Validation of power measurement techniques in dynamic lower body resistance exercises. J. Appl. Biomech..

[369] Lames M., McGarry T. (2007). On the search for reliable performance indicators in game sports. Int. J. Perform. Anal. Sport.

[370] James D.A., Lee J.B. (2016). The increasing adoption of consumer grade wearables: Comparing the apples and oranges of sport science. J. Fit. Res..

[371] Bergamini E., Ligorio G., Summa A., Vannozzi G., Cappozzo A., Sabatini A.M. (2014). Estimating orientation using magnetic and inertial sensors and different sensor fusion approaches: accuracy assessment in manual and locomotion tasks. Sensors.

[372] Picerno P., Cereatti A., Cappozzo A. (2011). A spot check for assessing static orientation consistency of inertial and magnetic sensing units. Gait Posture.

[373] Syed Z.F., Aggarwal P., Goodall C., Niu X., El-Sheimy N. (2007). A new multi-position calibration method for MEMS inertial navigation systems. Meas. Sci. Technol..

[374] Fong W.T., Ong S.K., Nee A.Y.C. (2008). Methods for in-field user calibration of an inertial measurement unit without external equipment. Meas. Sci. Technol..

[375] Olivares A., Olivares G., Gorriz J.M., Ramirez J. High-efficiency low-cost accelerometer-aided gyroscope calibration. Proceedings of the International Conference on Test and Measurement.

[376] Gebre-Egziabher D., Elkaim G.H., Powell J.D., Parkinson B.W. A non-linear, two-step estimation algorithm for calibrating solid-state strapdown magnetometers. Proceedings of the 8th International Conference on Integrated Navigation Systems.

[377] Lüken M., Misgeld B., Rüschen D., Leonhardt S. (2015). Multi-sensor calibration of low-cost magnetic, angular rate and gravity systems. Sensors.

[378] Winter D.A., Winter D. (1990). Biomechanics and Motor Control of Human Movement.

[379] De Vries W.H.K., Veeger H.E.J., Baten C.T.M., van der Helm F.C.T. (2009). Magnetic distortion in motion labs, implications for validating inertial magnetic sensors. Gait Posture.

